# Supplementary material for: ERASE-ing Patient Mistreatment of Trainees: Faculty Workshop
Source: MedEdPORTAL. 2019 Dec 27;15:10865. doi: 10.15766/mep_2374-8265.10865 (PMC7012314; doi:10.15766/mep_2374-8265.10865)
Supplement: Supplementary file 1 — A. Facilitator Guide.docx B. PowerPoint Presentation.pptx C. Case Examples.docx D. ERASE Model Handout.docx E. Available Resources and Reporting Mechanisms Handout.docx F. Pre- and Postsession Surveys.docx [file mep-15-10865-s001.zip › _Educational_Summary_Report_10865.pdf]

MedEdPORTAL® The AAMC Journal of Teaching and Learning Resources

Original Publication Open Access

ERASE-ing Patient Mistreatment of Trainees: Faculty Workshop

Kirsten M. Wilkins, MD*, Matthew N. Goldenberg, MD, MSc, Kali D. Cyrus, MD, MPH

*Corresponding author: kirsten.wilkins@yale.edu

Abstract

Introduction: Mistreatment of physicians by patients is a long-standing phenomenon that has garnered increased attention recently. Medical students and residents also experience mistreatment, and many supervising physicians do not know how to recognize it or respond appropriately. Little guidance exists as to how faculty should best address these situations. We developed, taught, and evaluated a stepwise approach to help faculty physicians manage patient mistreatment of trainees (residents and students). **Methods:** Our approach is summarized by the acronym ERASE: (1) Expect that mistreatment will occur. (2) Recognize episodes of mistreatment. (3) Address the situation in real time. (4) Support the learner after the event. (5) Establish/encourage a positive culture. We designed an interactive, case-based educator development session to teach ERASE and surveyed participants before and after to evaluate the session. Sixty-nine participants attended one of four workshops between November 2017 and January 2018. **Results:** Nearly 80% of attendees reported having received no prior training in managing mistreatment of trainees by patients. Participants noted significant changes in their confidence in recognizing and responding to episodes of mistreatment after the session compared with just prior to it. **Discussion:** ERASE fills an important void in medical education by introducing a novel, easy-to-understand approach that faculty can employ to manage mistreatment of trainees. We have continued to disseminate this model to faculty and residents in various departments around our medical center and at national conferences. This resource will allow educators to disseminate the ERASE model at their home institutions.

Keywords
Race, Gender, Learning Environment, Faculty Development, Diversity, Inclusion, Health Equity

Educational Objectives

By the end of this activity, learners will be able to:

1. Discuss the prevalence and impact of mistreatment by patients on trainees and the learning environment.
2. Describe the role of supervisors and the institution in monitoring and responding to mistreatment by patients and identify potential barriers to this process.
3. Contrast the meanings of and interventions for mistreatment by patients as opposed to mistreatment by supervisors, peers, or other staff.
4. Identify various forms of mistreatment by patients.
5. Apply at least three practical strategies for responding to mistreatment of trainees.

Citation:
Wilkins KM, Goldenberg MN, Cyrus KD. ERASE-ing patient mistreatment of trainees: faculty workshop. *MedEdPORTAL*. 2019;15:10865. https://doi.org/10.15766/mep_2374-8265.10865

Introduction

As part of routine educational surveillance at our institution, we regularly survey trainees about their experiences of mistreatment in clinical settings. Historically, students have primarily reported mistreatment by faculty, residents, nurses, hospital staff, or occasionally other students.[1,2] Over the past few years, we began to notice that students increasingly reported mistreatment by patients. Most often, these incidents involved verbal insults or slights. Students told us that their supervisors often did not recognize or address the mistreatment. They expressed concern that these incidents negatively impacted their learning.[3]

Recent national surveys have confirmed that patient mistreatment of physicians and other health care providers is common. Nearly 60% of physicians report having received biased comments from patients, and nearly 50% have had patients request a change in provider due to some physician demographic characteristic.[4] Although the impact of these incidents varies, half of the physicians who have faced such discriminatory comments/behaviors have experienced a moderate to strong emotional reaction.[5]

Copyright © 2019 Wilkins et al. This is an open-access publication distributed under the terms of the Creative Commons Attribution license.

Despite the increasing awareness of the problem, there unfortunately is scant guidance in the academic medicine literature on how to best manage these situations.[6] To our knowledge, no published resource exists in *MedEdPORTAL* or elsewhere to teach faculty how to do so. Few physicians have been trained to address these situations when they arise in their own practice; most faculty members have similarly little to no training in how to intervene when such events happen to their trainees. Conversations with colleagues around our medical center and at national educational organization meetings confirmed a lack of both a specific approach to managing mistreatment by patients and a method for teaching faculty how to respond. We sought to fill these gaps by developing and disseminating the ERASE framework through a variety of workshops (see our *Academic Psychiatry* publication[7] and below for further description), which were evaluated as described in the Methods section. This resource supplements our *Academic Psychiatry* publication by providing the explicit instructional details and materials needed to implement the workshop.

Methods

We developed a stepwise approach, known by the acronym ERASE, for faculty to manage the mistreatment of trainees by patients.

- Expect that such events will happen and prepare accordingly.
  - Educator development.
  - Talk/read/rehearse specific language.
  - Provide anticipatory guidance to trainees.
- Recognize the mistreatment.
  - Consider the perspective of the trainee.
  - Distinguish between types of mistreatment.
  - Pay particular attention to potential microaggressions, "compliments."
- Address the situation in real time.
  - Consider context, goal, and tone.
  - Use specific language/technique depending on the situation.
  - Maintain a professional demeanor.
- Support the learner after the event.
  - Ask learners how they experienced the event.
  - Listen and respond to concerns.
  - Engage in decision making about next steps.
- Establish/encourage a positive culture.
  - Express openness to hearing concerns.
  - Develop and disseminate institutional reporting mechanisms.
  - Consider policies, signage regarding nontolerance for mistreatment.

We designed an interactive workshop aimed at faculty and senior residents to teach the ERASE model. The content of these sessions is contained in the appendices. (Appendix A is a facilitator guide that describes the conduct of the session.) We started each session with 5-10 minutes of welcome and introduction, including reminding participants that virtually all physicians struggle with how to respond in the face of mistreatment by patients. We also acknowledged our own difficulties addressing this issue. We then assured participants that the session was a safe space for candid discussion and that we were all there to learn and improve skills as educators.

Workshop facilitators next led an interactive lecture, guided by PowerPoint slides (Appendix B), in which they reviewed background data on the prevalence/impact of mistreatment by patients and showed a YouTube video with personal narratives of physician mistreatment by patients.[8]

We asked participants to engage in 2-minute buzz-group discussion with their neighbors (slide 13): "What are the barriers to addressing mistreatment and harassment by patients in the clinical setting? Discuss with your neighbors for 2 minutes." After 2 minutes, we facilitated participants' sharing their responses with the larger group.

We then asked participants to engage in another 2-minute buzz-group discussion (slide 14): "How does addressing mistreatment and harassment by patients differ from that by colleagues? Discuss with your neighbors for 2 minutes." After 2 minutes, we facilitated participants' sharing their responses with the larger group.

Next, facilitators formally introduced the ERASE framework, including three common examples of mistreatment by patients and recommended interventions and sample language for each (slides 18-20) while acknowledging that this was not an exhaustive list of problematic verbal comments from patients. We encouraged participants to share additional problematic comments they had experienced. We reminded participants that choice of intervention and language used would depend on a variety of factors, including the individual's comfort level with the clinical situation, sense of safety, and personality. There was no one right way to respond in all situations.

Copyright © 2019 Wilkins et al. This is an open-access publication distributed under the terms of the Creative Commons Attribution license.

The remainder of the workshop (40 minutes) involved participants' practicing applying the ERASE model. We divided participants into small groups of three to five people each. We selected three cases from those provided in the session materials (Appendix C), one representing each of the three common problems (derogatory language, microaggression, "complimentary" comment). We assigned each small group one of the three practice cases such that the three cases were distributed evenly across the entire group. Small groups spent 10 minutes reviewing their assigned case and practicing applying the ERASE framework to each case as follows: (a) What type of mistreatment do you recognize? (b) How might the faculty member in the case address the situation in real time, including specific language to be used? (c) How might faculty support the learner, again identifying specific language? (d) What institutional interventions might be needed to establish/encourage a positive culture? We facilitated a discussion of each case by having volunteers from each small group share (a)-(d) with the larger group.

Session leaders then led a 5- to 10-minute wrap-up in which they summarized take-home points and reinforced the ERASE framework, distributed handouts with the ERASE framework and sample language (Appendix D), and provided a list of resources for support and available reporting mechanisms at our institution (Appendix E). Participants were encouraged to read Diane J. Goodman's "Responding to Biased or Offensive Comments,"[9] which was often circulated after the session by email as an additional resource.

We delivered the workshop in four different sessions (1-1.5 hours in length) around the academic medical center in late 2017 and early 2018: (1) clerkship educator development session (attended by 25 faculty and residents in psychiatry, internal medicine, and pediatrics), (2) on-site Veterans Affairs primary care center (11 faculty in internal medicine), (3) psychiatric hospital (16 psychiatry faculty), and (4) mental health center (17 psychiatry faculty and residents). Sessions were led by two to three author-facilitators. No presession preparation was required for participants.

To evaluate the workshop, we surveyed participants with a Likert-style questionnaire (1 = *strongly disagree*, 5 = *strongly agree*; Appendix F) about their attitudes and behaviors toward trainee mistreatment both immediately prior to and immediately following the workshop. We compiled the survey responses from participants in all sessions and analyzed them in the aggregate. We compared the results of each matched question of the pre- and postsurveys using a paired *t* test. We also solicited informal feedback after each session, asking participants to share their impressions and suggestions verbally and debriefing with other author-presenters about our experience with the session.

Results

A total of 69 participants attended a workshop and participated in the evaluations. Although the original survey included optional fields for race/ethnicity, gender, and age, many participants left one or more of these sections blank, making any subgroup analysis impossible.

Seventy-eight percent of participants had not previously received specific training on recognizing and addressing episodes of trainee mistreatment.

Participants demonstrated significant changes from before the session to after the session in their attitudes toward and perceived skills in addressing trainee mistreatment (see the Table). Following the session, participants more strongly agreed that mistreatment could have a significant psychological impact (4.5 vs. 4.2, $p < .016$). Participants also increasingly believed that it was their role to respond to episodes of mistreatment by patients (4.5 vs. 4.2, $p < .018$). They reported feeling more able to recognize various forms of mistreatment (4.3 vs. 3.7, $p < .0001$), more able to intervene during episodes of patient mistreatment (4.1 vs. 3.1, $p < .0001$), and that they had a standardized approach to addressing an episode of mistreatment (3.7 vs. 2.4, $p < .0001$). No difference was found in participants' opinion that they currently intervened effectively or that they fostered a psychologically safe environment for trainees.

Informal feedback on the sessions from participants and presenters alike was positive. Participants expressed keen interest in the topic, engaged actively in the discussion, and expressed gratitude for the opportunity to share their own experiences of mistreatment by patients and to learn and practice techniques (including specific wording) for addressing future episodes.

Discussion

ERASE training sought to fill a void in professional development of academic medicine faculty by raising consciousness of and providing skills to manage the mistreatment of trainees by patients. The 1.5-hour workshop described here increases faculty awareness of the problem as well as know-how and resolve to respond to future episodes of mistreatment.

We learned several lessons during our presentations. First, time management is critical. With significant audience participation, it is incumbent upon presenters to guide discussion and stay within

Copyright © 2019 Wilkins et al. This is an open-access publication distributed under the terms of the Creative Commons Attribution license.

Table. Pre- and Postsession Survey Results[a]

Statement	Presession (n = 63)[a]	Postsession (n = 69)[a]	Change (95% CI)[b]	p
Mistreatment . . . is common . . . can have significant psychological impact.	4.2	4.5	0.33 (0.07 to 0.60)	.0152[c]
I intervene effectively when I learn of an episode of mistreatment.	3.4	3.7	0.25 (−0.03 to 0.53)	.0790
I foster a psychologically safe environment for my trainees.	4.1	4.0	−0.11 (−0.33 to 0.11)	.3299
It is my role to intervene when . . . a trainee under my supervision has been mistreated by a patient.	4.2	4.5	0.27 (0.05 to 0.49)	.0178[c]
It is my role to intervene when . . . a trainee under my supervision has been mistreated by a member of the health care team.	4.2	4.6	0.36 (0.14 to 0.59)	.0019[c]
I can recognize multiple forms of trainee mistreatment, including abuse, harassment, microaggressions.	3.7	4.3	0.56 (0.33 to 0.88)	<.0001[c]
I know how to intervene when I learn of an episode of trainee mistreatment by patients.	3.1	4.1	1.05 (0.79 to 1.29)	<.0001[c]
I know how to intervene when I learn of an episode of trainee mistreatment by a member of the health care team.	3.1	4.1	1.00 (0.75 to 1.26)	<.0001[c]
I have a standardized approach I can utilize when addressing an episode of trainee mistreatment.	2.4	3.7	1.29 (0.97 to 1.62)	<.0001[c]

Abbreviation: CI, confidence interval.
[a]Pre- and postsession values represent means of responses on a 5-point Likert scale (1 = *strongly disagree*, 2 = *disagree*, 3 = *neutral*, 4 = *agree*, 5 = *strongly agree*).
[b]Change values represent difference in means from pre- to postsession.
[c]$p < .05$.

predetermined time limits to complete the presentation. Second, presenters should expect a range of familiarity and comfort with the topics. Some in the audience may not be familiar, for example, with microaggressions, and some may be resistant to the idea that the described patient behaviors are even problematic. Third, we learned that at least one of our case examples could be interpreted as offensive. We described a patient's assumption that a female physician was a nurse as a microaggression. Some participants noted that there was nothing wrong with being a nurse, which obviously is true. We explained that the gendered assumption was the problem in the interaction. Perhaps our most important lesson, which affirms our work, was how high the demand for this content is.

The content of the workshop has resonated both locally and nationally—since introducing ERASE at our institution and at several national meetings (American Association of Directors of Psychiatric Residency Training 2018, Association of Directors of Medical Student Education in Psychiatry 2018, Association of American Medical Colleges 2018), numerous faculty from different departments and at peer institutions have asked us to provide materials and instructions to help them offer similar trainings. This publication provides these materials and guidance, including how the session might be adapted for different time (e.g., 1 hour) or format (e.g., grand rounds) constraints. (See the facilitator guide in Appendix A for suggestions.)

Although the model and workshop were initially designed to teach medical school faculty to respond to patient mistreatment, we have fielded inquiries about adapting the model to teach and empower trainees or nonsupervising physicians directly (i.e., shifting from "What should I do as a bystander to mistreatment?" to "What should I do as a recipient of mistreatment?"). We have also collaborated with other disciplines, including the nursing department at Yale New Haven Children's Hospital, to adapt the training to include nonphysician health care providers. In these cases, the S of ERASE becomes "Seek support from a colleague, trusted mentor, or supervisor," and cases and sample responses can be adapted accordingly.

There are several limitations to our study. We measured only awareness of the problem of mistreatment and confidence in addressing it but did not measure participants' actual knowledge or skills. Furthermore, we do not know whether participants' behavior changed in recognizing and addressing episodes of mistreatment in clinical settings. We also do not know their experience in implementing the model in vivo. Because our surveys were conducted immediately prior to and following the didactic session, we do not know if any changes in confidence, knowledge, or skills were maintained over time. Finally, the same three authors presented to psychiatric and primary care faculty and residents at one academic medical center, so we do not know whether other specialties will be as receptive or whether the approach will work at other institutions or with different presenters.

Future study should include whether and to what extent workshop participants implement ERASE in the clinical setting, what their experiences are, and whether the immediate benefits of this workshop are maintained over time. Disseminating this work to other departments and other institutions will allow for studying the broader relevance, acceptability, and effectiveness of this model. Adapting the model for use by other disciplines (e.g., nursing, social work) and by trainees themselves to empower them to respond directly as recipients of mistreatment is another future direction.

We are hopeful that the ERASE model and our detailed training guide will be useful to educators and managers looking to

Copyright © 2019 Wilkins et al. This is an open-access publication distributed under the terms of the Creative Commons Attribution license.

combat the scourge of patient mistreatment of health care providers, including medical students and residents.

Appendices

A. Facilitator Guide.docx

B. PowerPoint Presentation.pptx

C. Case Examples.docx

D. ERASE Model Handout.docx

E. Available Resources and Reporting Mechanisms Handout.docx

F. Pre- and Postsession Surveys.docx

All appendices are peer reviewed as integral parts of the Original Publication.

Kirsten M. Wilkins, MD: Associate Professor, Department of Psychiatry, Yale University School of Medicine

Matthew N. Goldenberg, MD, MSc: Associate Professor, Department of Psychiatry, Yale University School of Medicine

Kali D. Cyrus, MD, MPH: Assistant Professor, Department of Psychiatry and Behavioral Sciences, Johns Hopkins University School of Medicine

Acknowledgments
The authors wish to thank the Department of Psychiatry and the Teaching and Learning Center at the Yale University School of Medicine.

Disclosures
None to report.

Funding/Support
None to report.

Prior Presentations

Presentations

Cyrus KD, Rohrbaugh RM. Speaking up for students: ERASE-ing mistreatment by patients. Presented at: American Association of Directors of Psychiatric Residency Training Annual Meeting; March 1-3, 2018; New Orleans, LA.

Wilkins KM, Goldenberg MN, Sebastian D. Speaking up for students: ERASE-ing mistreatment by patients. Presented at: Association of Directors of Medical Student Education in Psychiatry Annual Meeting; June 14-16, 2018; Minneapolis, MN.

Wilkins KM, Cyrus KD, Goldenberg MN. Speaking up for students: how to recognize and address mistreatment by patients. Presented at: Learn Serve Lead: The Annual AAMC Meeting; November 2-6, 2018; Austin, TX.

Publication

Goldenberg MN, Cyrus KD, Wilkins KM. ERASE: a new framework for faculty to manage patient mistreatment of trainees. *Acad Psychiatry.* 2019;43(4):396-399. https://doi.org/10.1007/s40596-018-1011-6

Ethical Approval

The Yale Institutional Review Board approved this study.

References

1. Wood DF. Bullying and harassment in medical schools. *BMJ*. 2006;333(7570):664-665. https://doi.org/10.1136/bmj.38954.568148.BE
2. Mavis B, Sousa A, Lipscomb W, Rappley MD. Learning about medical student mistreatment from responses to the Medical School Graduation Questionnaire. *Acad Med*. 2014;89(5): 705-711. https://doi.org/10.1097/ACM.0000000000000199
3. Cyrus KD, Angoff NR, Illuzzi JL, Schwartz ML, Wilkins KM. When patients hurt us. *Med Teach*. 2018;40(12):1308-1309. https://doi.org/10.1080/0142159X.2018.1428291
4. Watson S. Credentials don't shield doctors, nurses from bias. WebMD website. https://www.webmd.com/a-to-z-guides/news/20171018/survey-patient-bias-toward-doctors-nurses. Published October 18, 2017. Accessed February 12, 2019.
5. Dvir Y, Moniwa E, Crisp-Han H, Levy D, Coverdale JH. Survey of threats and assaults by patients on psychiatry residents. *Acad Psychiatry*. 2012;36(1):39-42. https://doi.org/10.1176/appi.ap.10060090
6. Whitgob EE, Blankenburg RL, Bogetz AL. The discriminatory patient and family: strategies to address discrimination towards trainees. *Acad Med*. 2016;91(11):S64-S69. https://doi.org/10.1097/ACM.0000000000001357
7. Goldenberg MN, Cyrus KD, Wilkins KM. ERASE: a new framework for faculty to manage patient mistreatment of trainees. *Acad Psychiatry*. 2019;43(4):396-399. https://doi.org/10.1007/s40596-018-1011-6
8. WebMD. Patient prejudice [video]. YouTube website. https://www.youtube.com/watch?v=m_Y_4romD2U. Published October 17, 2017. Accessed February 22, 2019.
9. Goodman DJ. Responding to biased or offensive comments. Diane J. Goodman, Ed.D., website. http://www.dianegoodman.com/documents/RespondingToBiasedOrOffensive-Commentsexcerptarticle.pdf. Adapted from: Goodman DJ. *Promoting Diversity and Social Justice: Educating People From Privileged Groups*. 2nd ed. New York, NY: Routledge; 2011.

Received: February 13, 2019
Accepted: August 17, 2019
Published: December 27, 2019

Copyright © 2019 Wilkins et al. This is an open-access publication distributed under the terms of the Creative Commons Attribution license.
