# Supplementary material for: ERASE-ing Patient Mistreatment of Trainees: Faculty Workshop
Source: MedEdPORTAL. 2019 Dec 27;15:10865. doi: 10.15766/mep_2374-8265.10865 (PMC7012314; doi:10.15766/mep_2374-8265.10865)
Supplement: Supplementary file 1 — A. Facilitator Guide.docx B. PowerPoint Presentation.pptx C. Case Examples.docx D. ERASE Model Handout.docx E. Available Resources and Reporting Mechanisms Handout.docx F. Pre- and Postsession Surveys.docx [file mep-15-10865-s001.zip › A. Facilitator Guide.docx]

**ERASE-ing Patient Mistreatment of Trainees: Facilitator Guide**

# Total session time: 1.5 hours

# Ideal number of participants: 20-25

# (See below for suggested modifications if given a shorter time frame and larger audience.)

# Educational Objectives

By the end of this activity, learners will be able to:

1. Discuss the prevalence and impact of mistreatment by patients on trainees and the learning environment.
2. Describe the role of supervisors and the institution in monitoring and responding to mistreatment by patients, and identify potential barriers to this process.
3. Contrast the meaning(s) and intervention(s) for mistreatment by patients as opposed to mistreatment by supervisors, peers, or other staff.
4. Recognize various forms of mistreatment by patients.
5. Apply at least 3 practical strategies for responding to mistreatment of trainees.

# Materials

# PowerPoint slide presentation

# Discussion cases

# “ERASE” handout

# “Examples of interpersonal interventions” handout

# “Available resources and reporting mechanisms” handout

# Session Format

1. **Welcome and introductions (5-10 minutes)**
   1. Remind participants that virtually all physicians struggle with how to respond in the face of mistreatment by patients. Facilitators may wish to acknowledge their own difficulties addressing this issue.
   2. Review “ground rules”: this is a safe space for candid discussion. All are here to learn and improve skills as educators.
2. **Interactive opening lecture (30 minutes)**
   1. Review background data on prevalence/impact of mistreatment by patients. Show YouTube video with personal narratives of physician mistreatment by patients.
   2. Ask participants to engage in 2-minute buzz group discussion (slide 13): “What are the barriers to addressing mistreatment and harassment by patients in the clinical setting? Discuss with your neighbors for 2 minutes.” After 2 minutes, facilitate participants’ sharing their responses with the larger group. Common responses include:
      1. Lack of time
      2. Not knowing how to intervene or what to say
      3. Not recognizing that mistreatment has occurred
      4. Fear of damaging the therapeutic alliance
      5. Fear of escalating the situation
      6. Fear that intervention will not be supported by the institution (e.g., hospital/clinic culture of “the patient is always right”) or will negatively impact the institution (e.g., Press-Ganey scores)
   3. Ask participants to engage in 2-minute buzz group discussion (slide 14): “How does addressing mistreatment and harassment by patients differ from that by colleagues? Discuss with your neighbors for 2 minutes.” After 2 minutes, facilitate participants’ sharing their responses with the larger group. Common responses include:
      1. More clearly defined reporting mechanisms and legal repercussions for mistreatment and harassment by colleagues
      2. Differences in power dynamics
      3. Often have longitudinal relationship with colleagues, may not be true for patients
   4. Introduction of ERASE framework, including 3 common examples of mistreatment by patients and recommended intervention/sample language for each (slides 18-20).
      1. Acknowledge this is not an exhaustive list of problematic verbal comments from patients.
      2. Encourage participants to share additional problematic comments they have experienced.
      3. Remind participants that choice of intervention and language used will depend on a variety of factors including the individual’s comfort level with the clinical situation, sense of safety, and personality. *There is no one right way to respond in all situations.*
3. **Skill practice (40 minutes)**
   1. Divide participants into small groups of 3-5 people each.
   2. Select 3 cases from those provided in session materials, one representing each of the 3 common problems (derogatory language, microaggression, “complimentary” comment). Assign each group 1 of the 3 practice cases, such that the 3 cases are distributed evenly across the entire group.
   3. Give groups 10 minutes to review their assigned case and practice applying the ERASE framework to each case as follows:
      1. What type of mistreatment do you recognize?
      2. How might the faculty member in the case address the situation in real time? (suggest specific language to be used)
      3. How might faculty support the learner? (again, ask for specific language)
      4. What institutional interventions might be needed to establish/encourage a positive culture?
   4. Facilitate discussion of each case, by having volunteers from each group share i-iv above with the larger group.
4. **Wrap Up (5-10 minutes)**
   1. Summarize take-home points/reinforce ERASE framework.
   2. Distribute handouts with ERASE framework and sample language.
   3. Encourage participants to read Diane Goodman’s “Responding to Biased or Offensive Comments”, which may be circulated via email after the session.
   4. Provide list of resources for support and available reporting mechanisms at your institution.

**Session Modifications**

This session may be modified for use with a non-faculty audience (e.g., trainees, nursing staff).

1. The “S” of ERASE becomes “Seek support from a colleague, trusted mentor, or supervisor.”
2. Cases and sample responses should be adapted accordingly.

This session may be conducted in 1 hour and with a larger group (e.g., Grand Rounds).

1. Eliminate educational objective #3 and the second buzz group slide on comparing mistreatment by patients to that by colleagues.
2. Eliminate one or more of the background data slides or narrative video.
3. Instead of breaking into small groups and distributing paper cases, project 1-2 cases to the entire group on slides. Use buzz group format to have participants discuss with their neighbors how they might apply ERASE to the case for 3-5 minutes. Then facilitate discussion with the larger group.
