# Supplementary material for: ERASE-ing Patient Mistreatment of Trainees: Faculty Workshop
Source: MedEdPORTAL. 2019 Dec 27;15:10865. doi: 10.15766/mep_2374-8265.10865 (PMC7012314; doi:10.15766/mep_2374-8265.10865)
Supplement: Supplementary file 1 — A. Facilitator Guide.docx B. PowerPoint Presentation.pptx C. Case Examples.docx D. ERASE Model Handout.docx E. Available Resources and Reporting Mechanisms Handout.docx F. Pre- and Postsession Surveys.docx [file mep-15-10865-s001.zip › B. PowerPoint Presentation.pptx]

## Slide 1
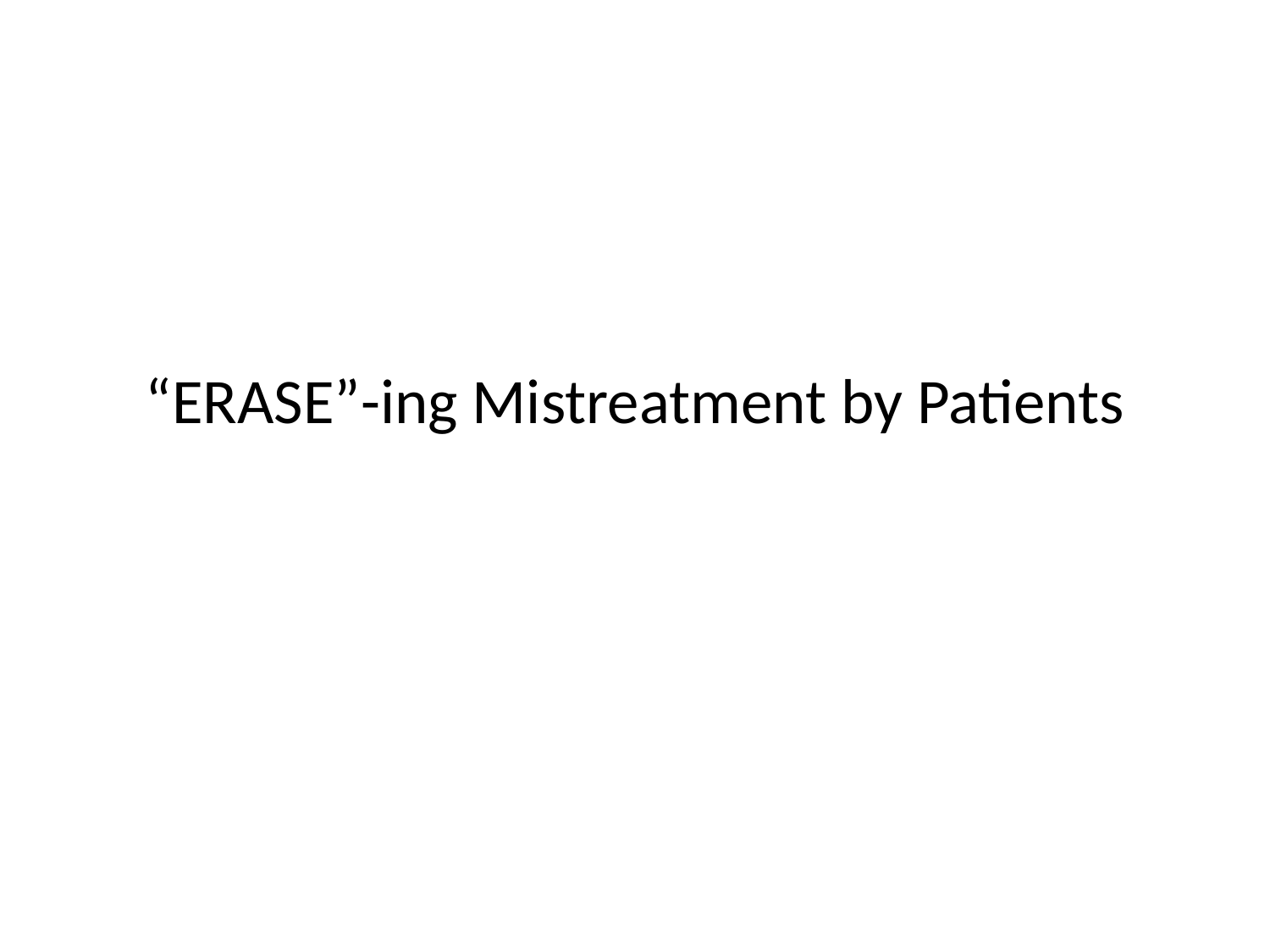

# “ERASE”-ing Mistreatment by Patients

## Slide 2
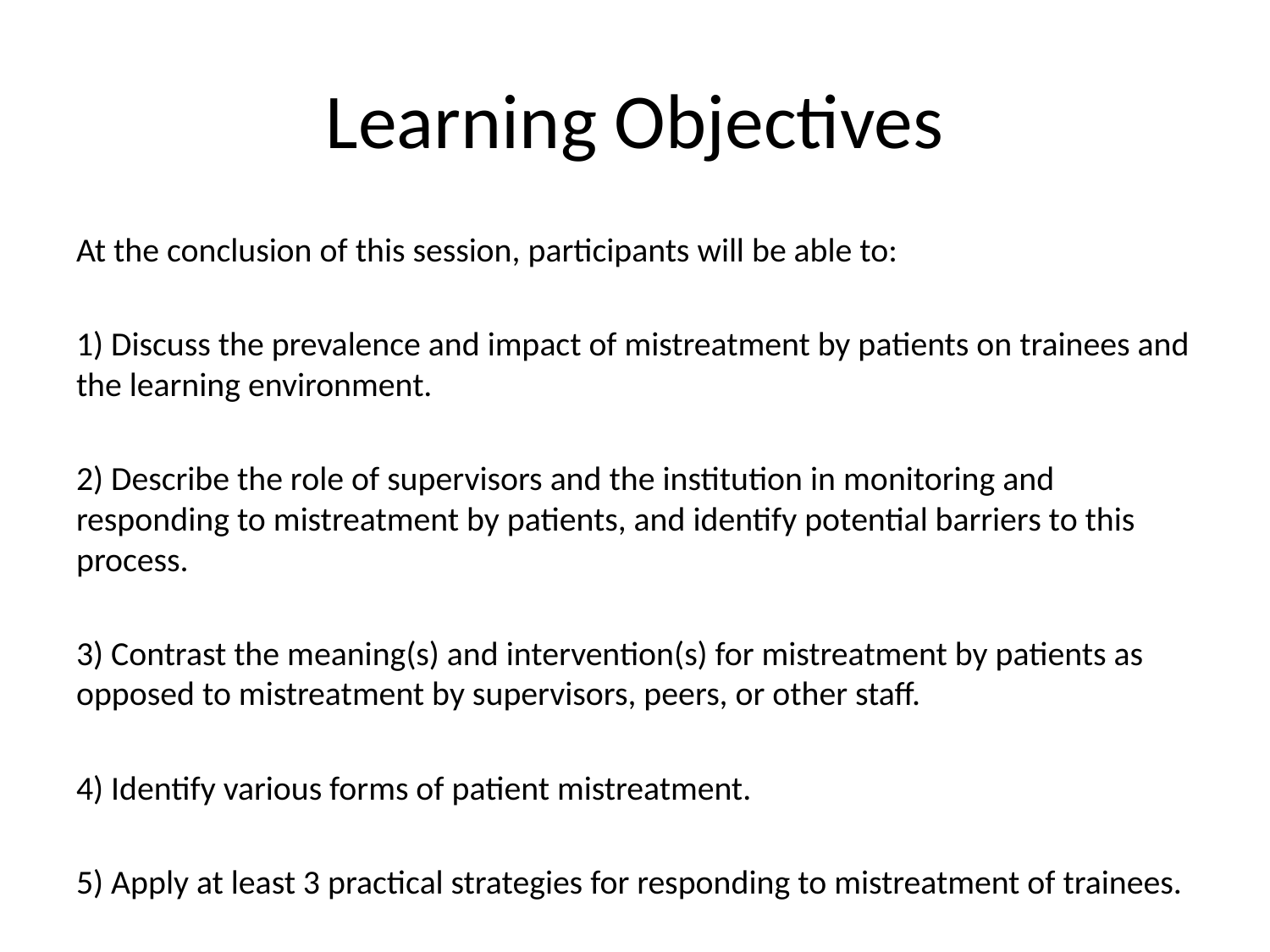

# Learning Objectives
At the conclusion of this session, participants will be able to:
1) Discuss the prevalence and impact of mistreatment by patients on trainees and the learning environment.
2) Describe the role of supervisors and the institution in monitoring and responding to mistreatment by patients, and identify potential barriers to this process.
3) Contrast the meaning(s) and intervention(s) for mistreatment by patients as opposed to mistreatment by supervisors, peers, or other staff.
4) Identify various forms of patient mistreatment.
5) Apply at least 3 practical strategies for responding to mistreatment of trainees.

## Slide 3
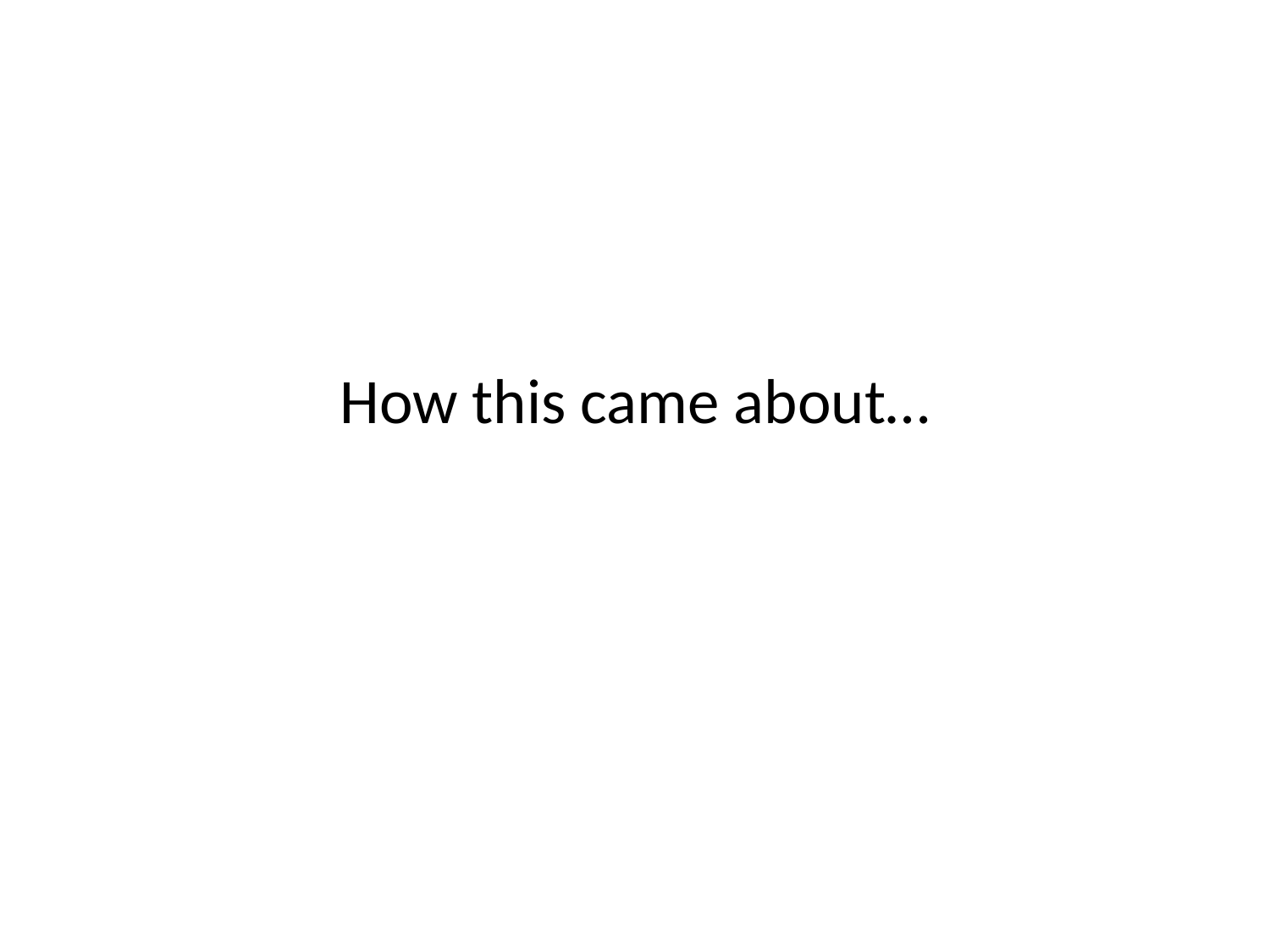

# How this came about…

## Slide 4
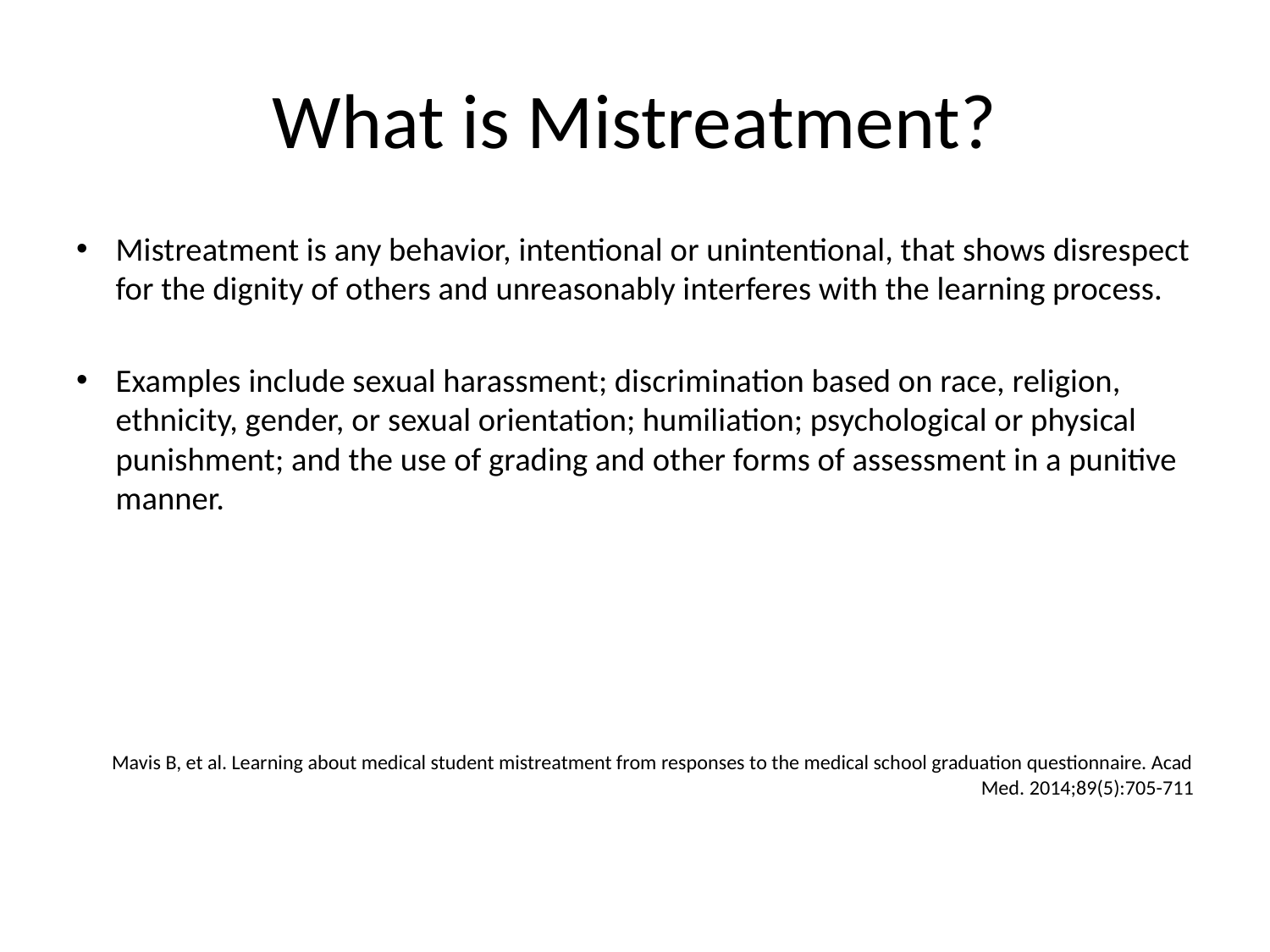

# What is Mistreatment?
Mistreatment is any behavior, intentional or unintentional, that shows disrespect for the dignity of others and unreasonably interferes with the learning process.
Examples include sexual harassment; discrimination based on race, religion, ethnicity, gender, or sexual orientation; humiliation; psychological or physical punishment; and the use of grading and other forms of assessment in a punitive manner.
Mavis B, et al. Learning about medical student mistreatment from responses to the medical school graduation questionnaire. Acad Med. 2014;89(5):705-711

## Slide 5
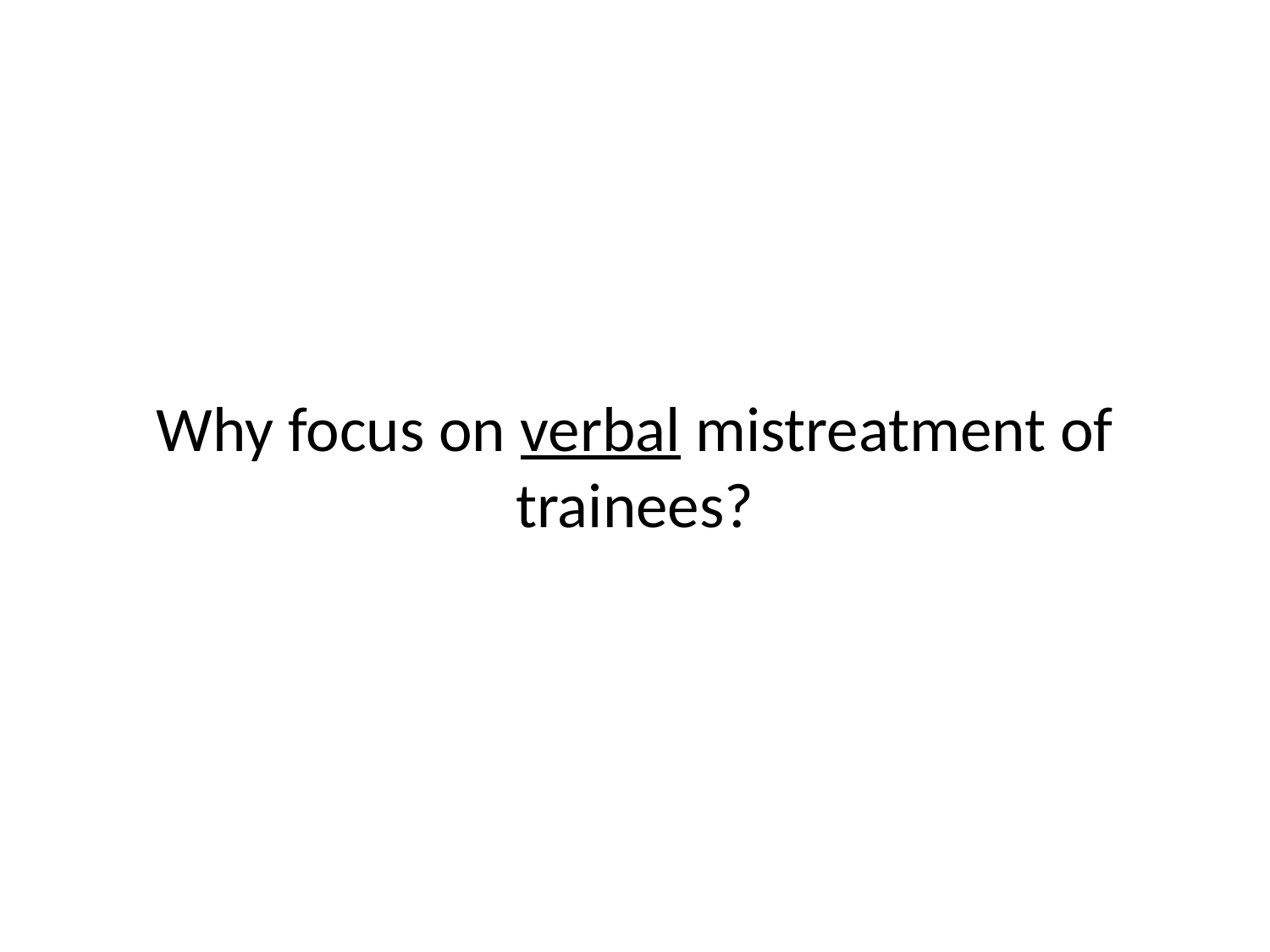

Why focus on verbal mistreatment of trainees?

## Slide 6
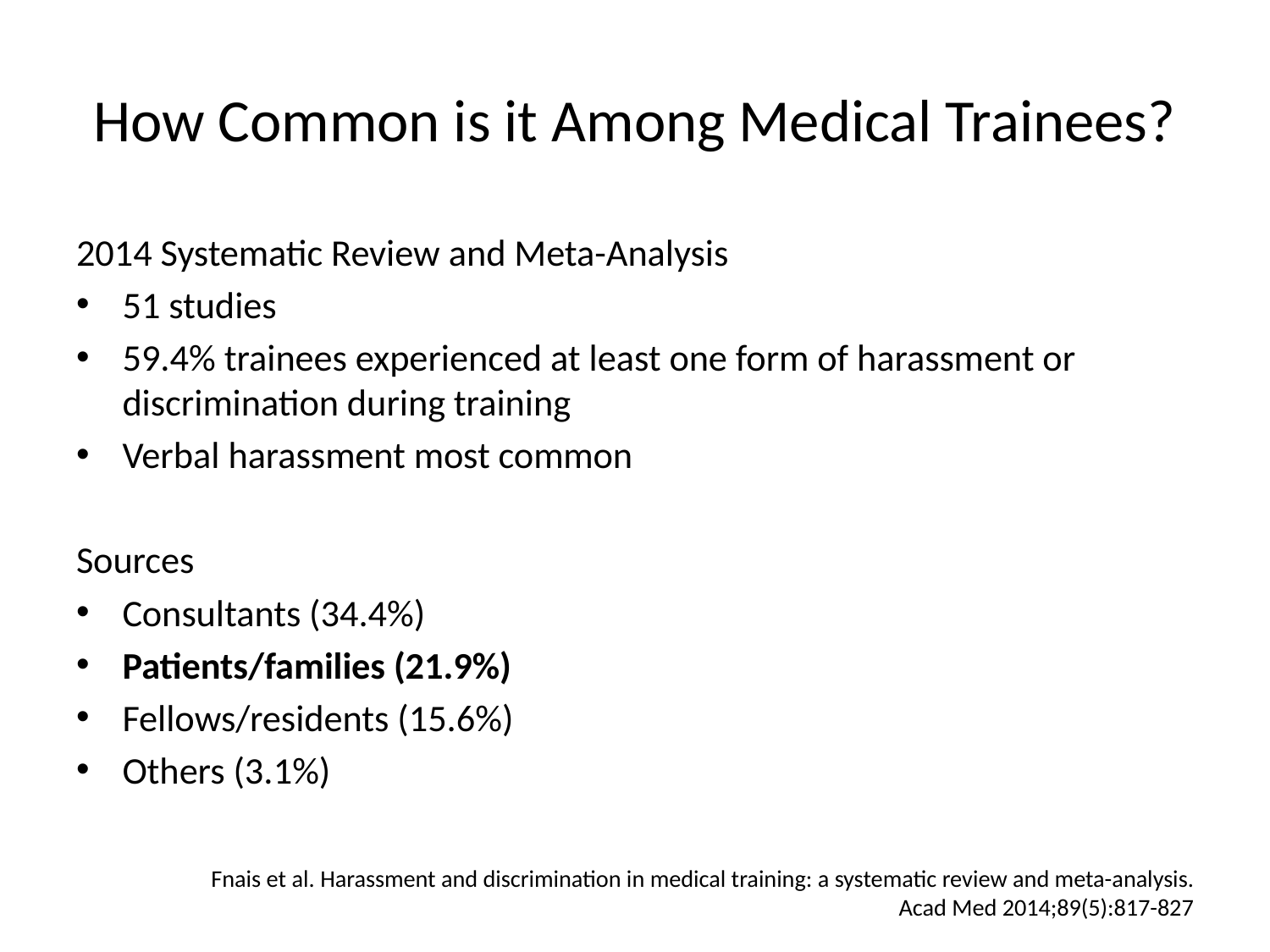

# How Common is it Among Medical Trainees?
2014 Systematic Review and Meta-Analysis
51 studies
59.4% trainees experienced at least one form of harassment or discrimination during training
Verbal harassment most common
Sources
Consultants (34.4%)
Patients/families (21.9%)
Fellows/residents (15.6%)
Others (3.1%)
Fnais et al. Harassment and discrimination in medical training: a systematic review and meta-analysis. Acad Med 2014;89(5):817-827

## Slide 7
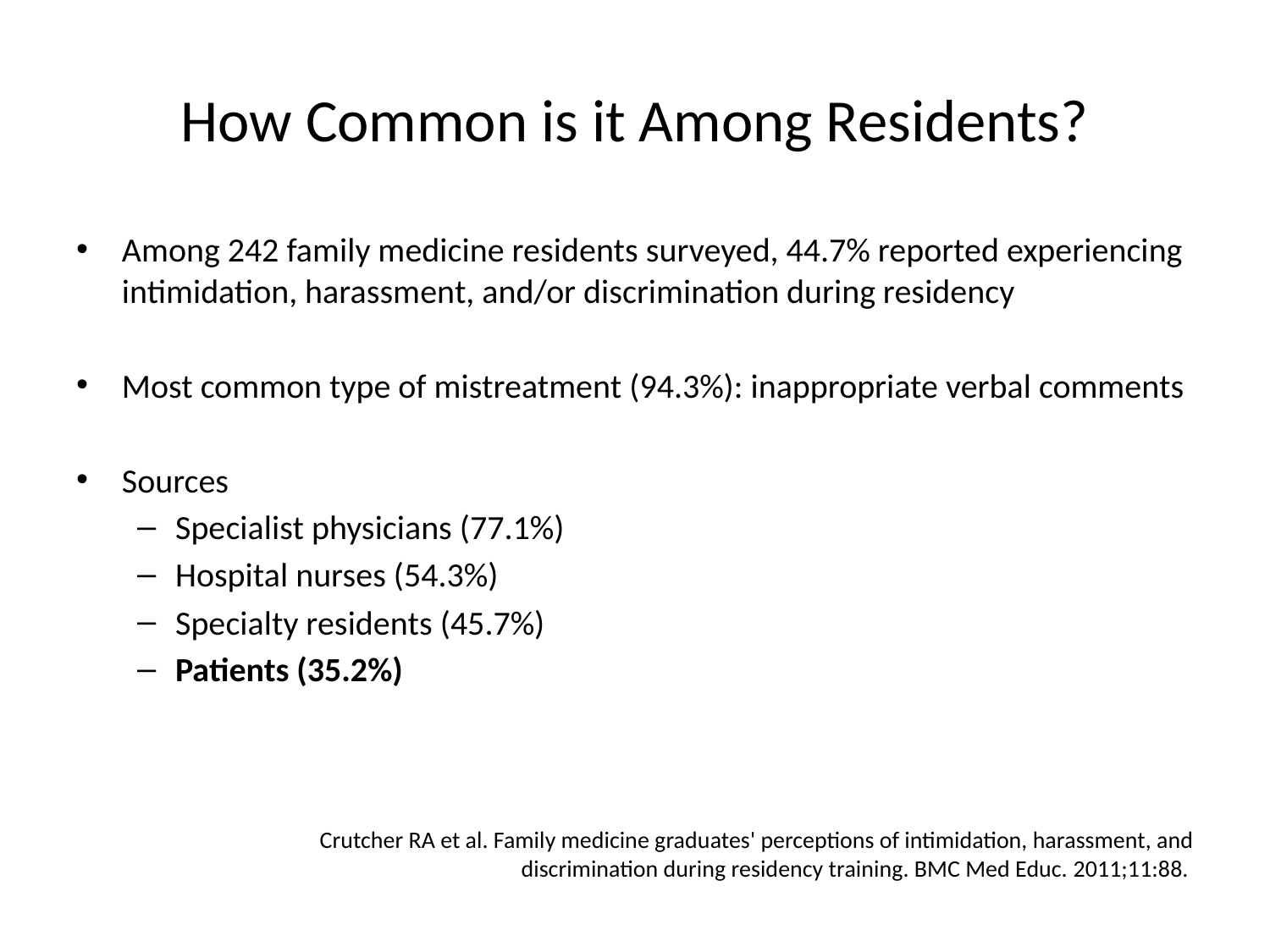

# How Common is it Among Residents?
Among 242 family medicine residents surveyed, 44.7% reported experiencing intimidation, harassment, and/or discrimination during residency
Most common type of mistreatment (94.3%): inappropriate verbal comments
Sources
Specialist physicians (77.1%)
Hospital nurses (54.3%)
Specialty residents (45.7%)
Patients (35.2%)
Crutcher RA et al. Family medicine graduates' perceptions of intimidation, harassment, and discrimination during residency training. BMC Med Educ. 2011;11:88.

## Slide 8
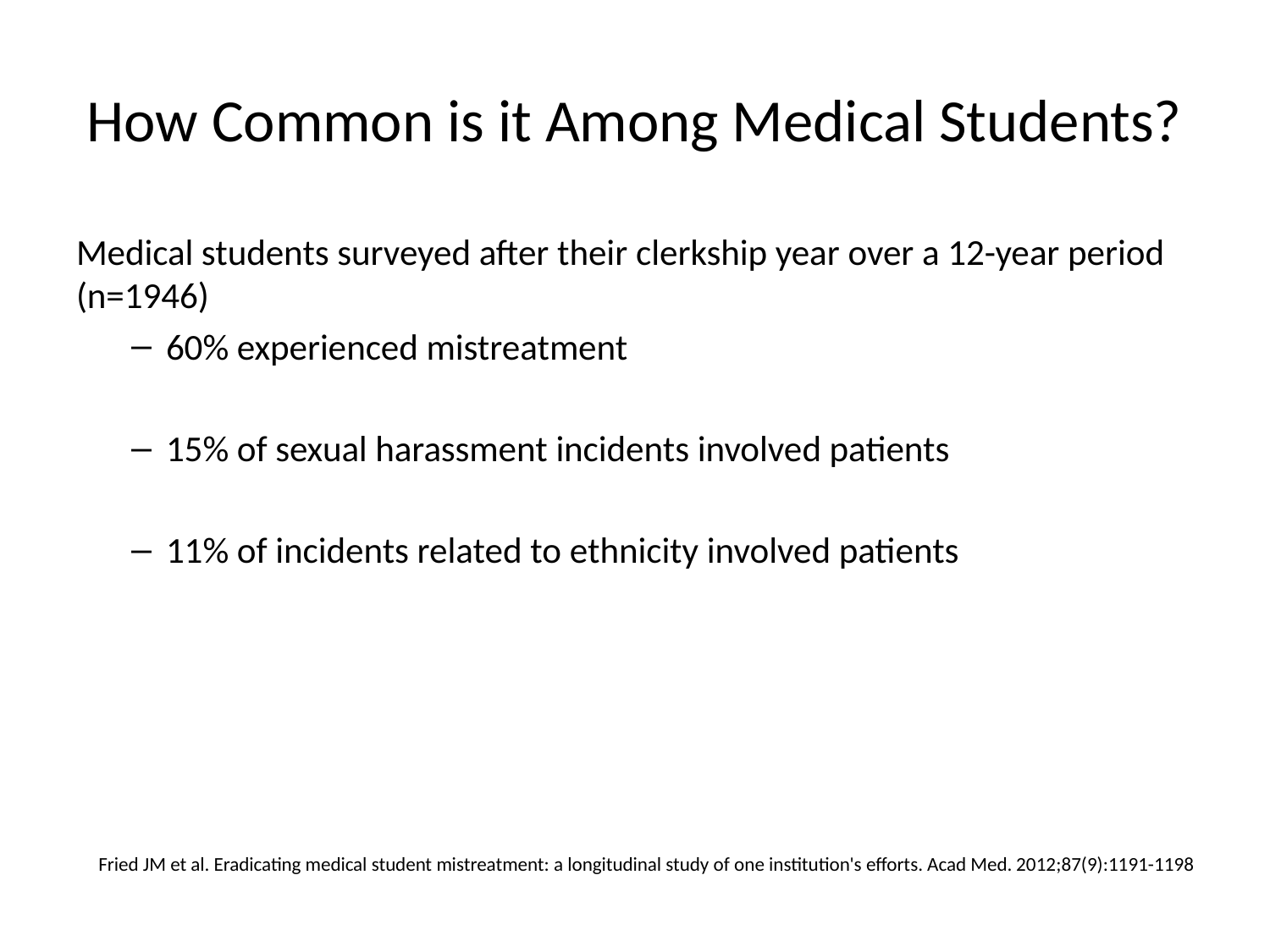

# How Common is it Among Medical Students?
Medical students surveyed after their clerkship year over a 12-year period (n=1946)
60% experienced mistreatment
15% of sexual harassment incidents involved patients
11% of incidents related to ethnicity involved patients
Fried JM et al. Eradicating medical student mistreatment: a longitudinal study of one institution's efforts. Acad Med. 2012;87(9):1191-1198

## Slide 9
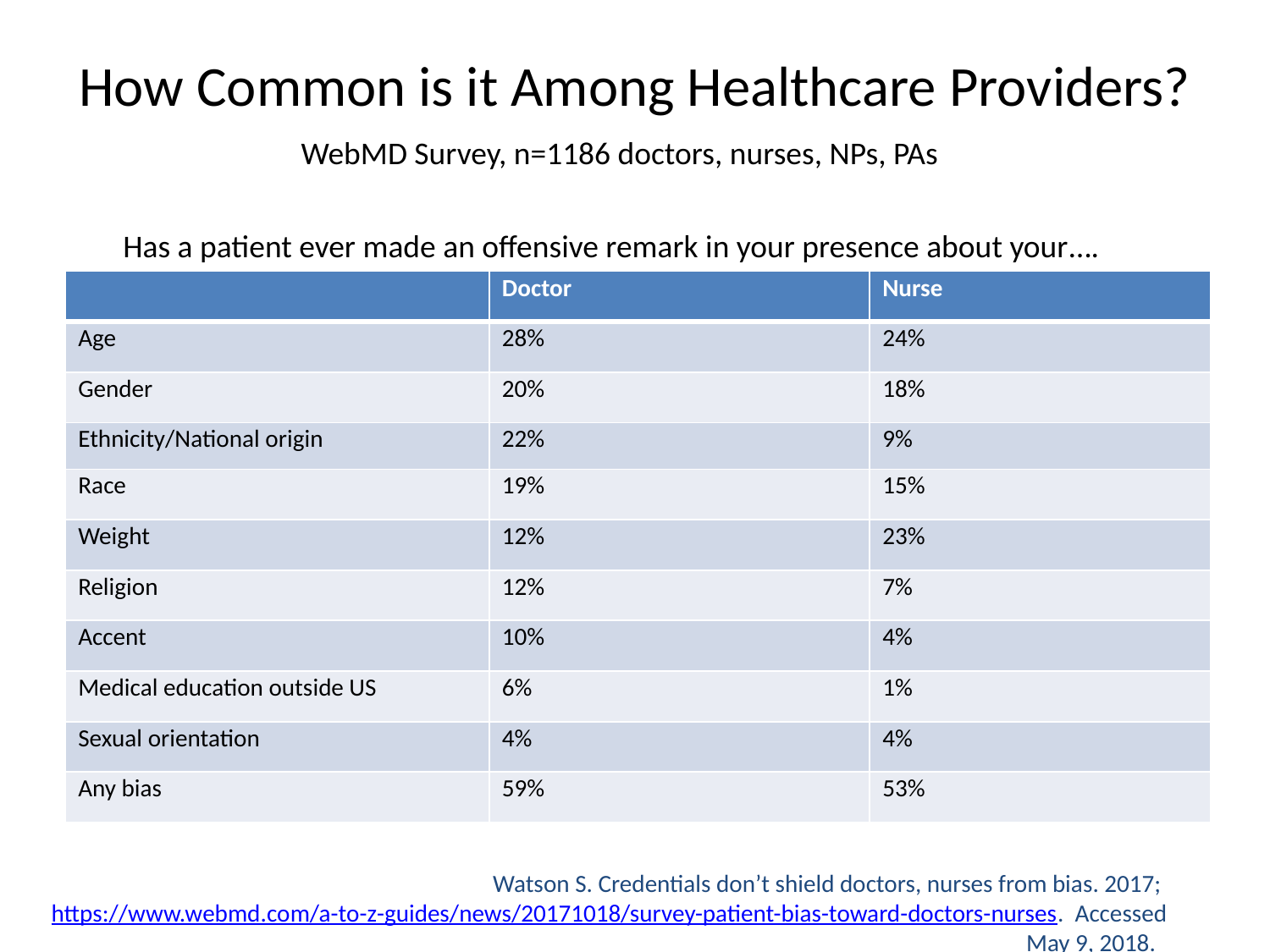

# How Common is it Among Healthcare Providers?
WebMD Survey, n=1186 doctors, nurses, NPs, PAs
Has a patient ever made an offensive remark in your presence about your….
| | Doctor | Nurse |
| --- | --- | --- |
| Age | 28% | 24% |
| Gender | 20% | 18% |
| Ethnicity/National origin | 22% | 9% |
| Race | 19% | 15% |
| Weight | 12% | 23% |
| Religion | 12% | 7% |
| Accent | 10% | 4% |
| Medical education outside US | 6% | 1% |
| Sexual orientation | 4% | 4% |
| Any bias | 59% | 53% |
Watson S. Credentials don’t shield doctors, nurses from bias. 2017; https://www.webmd.com/a-to-z-guides/news/20171018/survey-patient-bias-toward-doctors-nurses. Accessed May 9, 2018.

## Slide 10
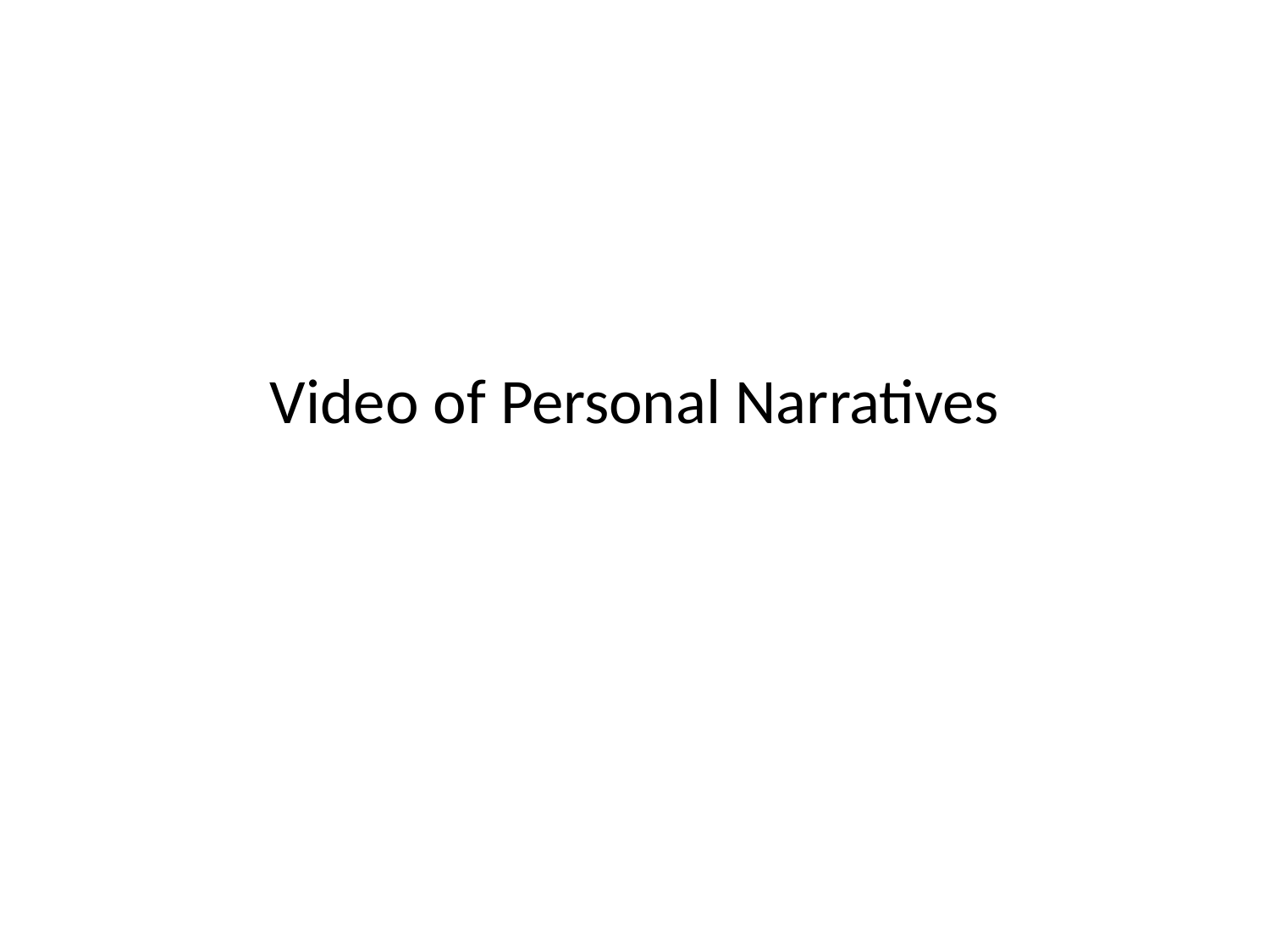

# Video of Personal Narratives

## Slide 11
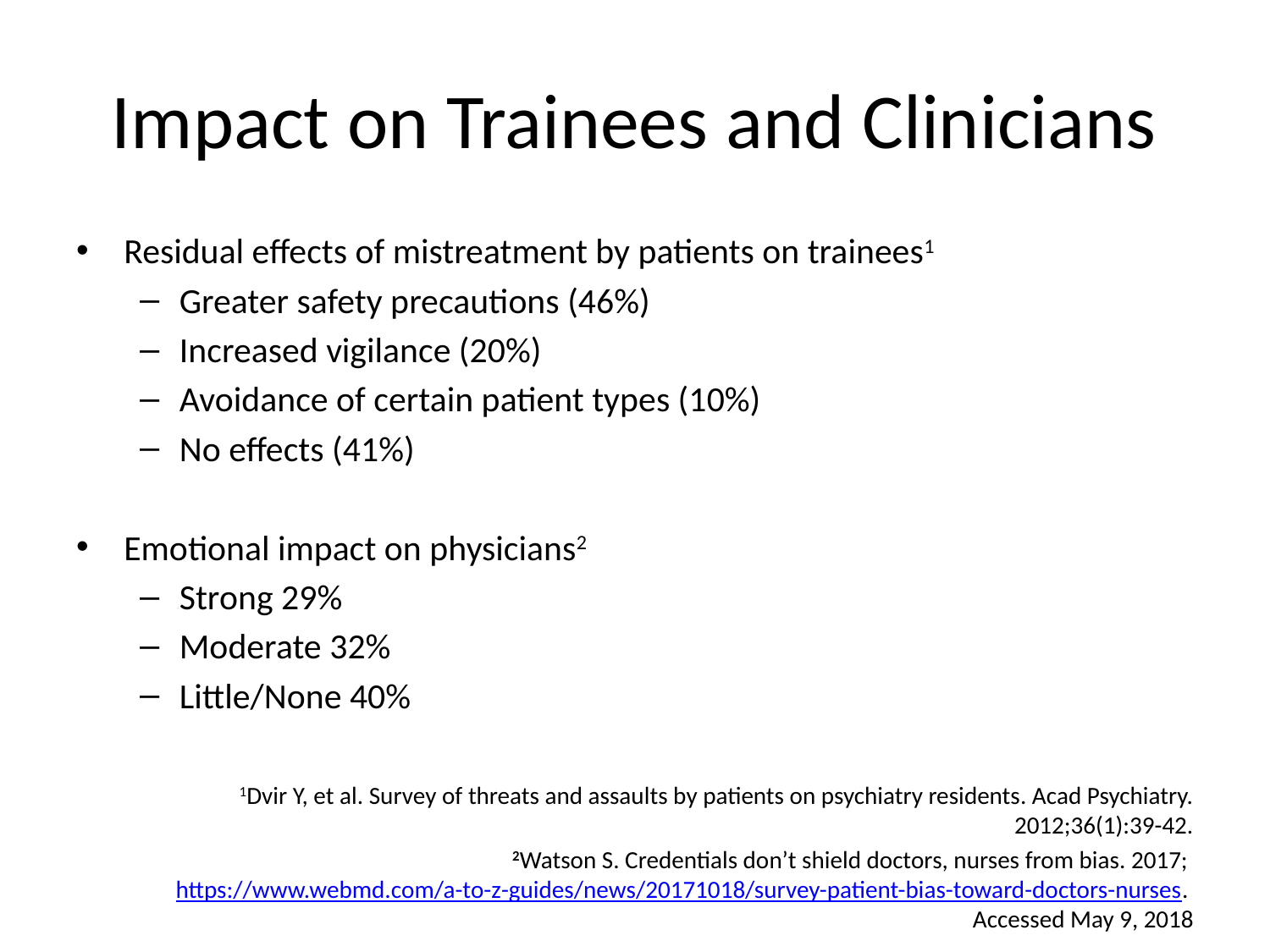

# Impact on Trainees and Clinicians
Residual effects of mistreatment by patients on trainees1
Greater safety precautions (46%)
Increased vigilance (20%)
Avoidance of certain patient types (10%)
No effects (41%)
Emotional impact on physicians2
Strong 29%
Moderate 32%
Little/None 40%
1Dvir Y, et al. Survey of threats and assaults by patients on psychiatry residents. Acad Psychiatry. 2012;36(1):39-42.
	2Watson S. Credentials don’t shield doctors, nurses from bias. 2017; https://www.webmd.com/a-to-z-guides/news/20171018/survey-patient-bias-toward-doctors-nurses. Accessed May 9, 2018

## Slide 12
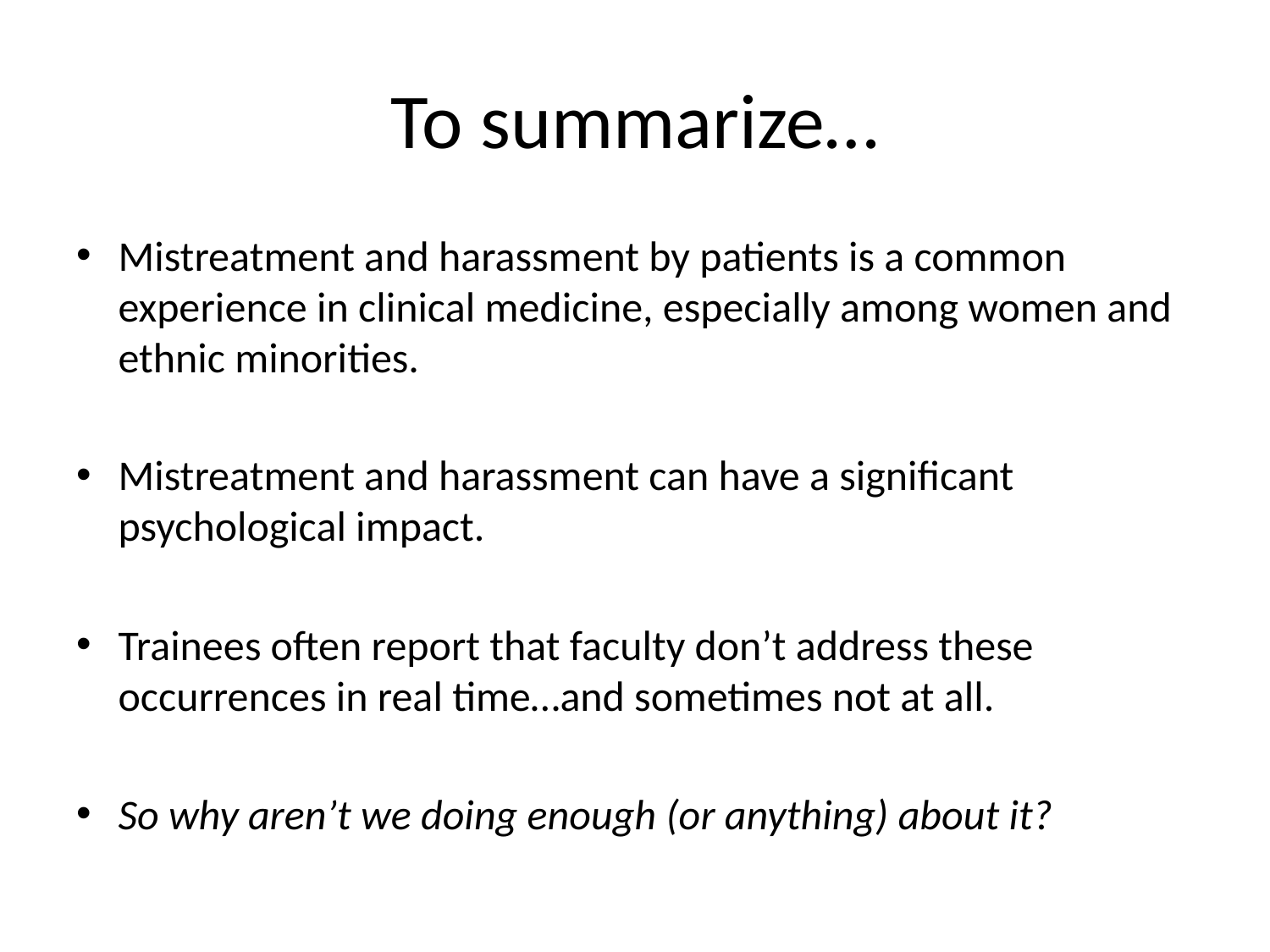

# To summarize…
Mistreatment and harassment by patients is a common experience in clinical medicine, especially among women and ethnic minorities.
Mistreatment and harassment can have a significant psychological impact.
Trainees often report that faculty don’t address these occurrences in real time…and sometimes not at all.
So why aren’t we doing enough (or anything) about it?

## Slide 13
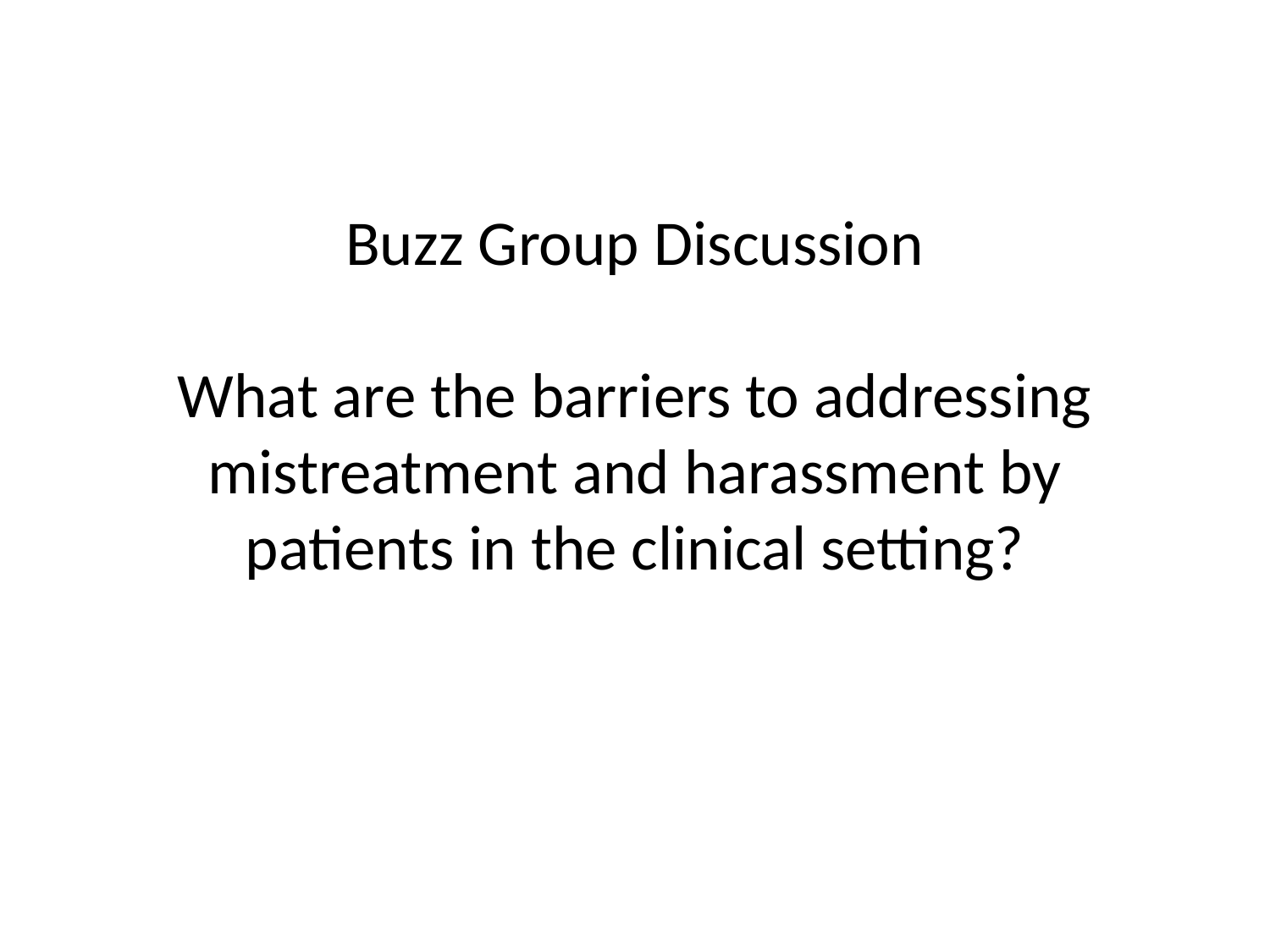

# Buzz Group Discussion What are the barriers to addressing mistreatment and harassment by patients in the clinical setting?

## Slide 14
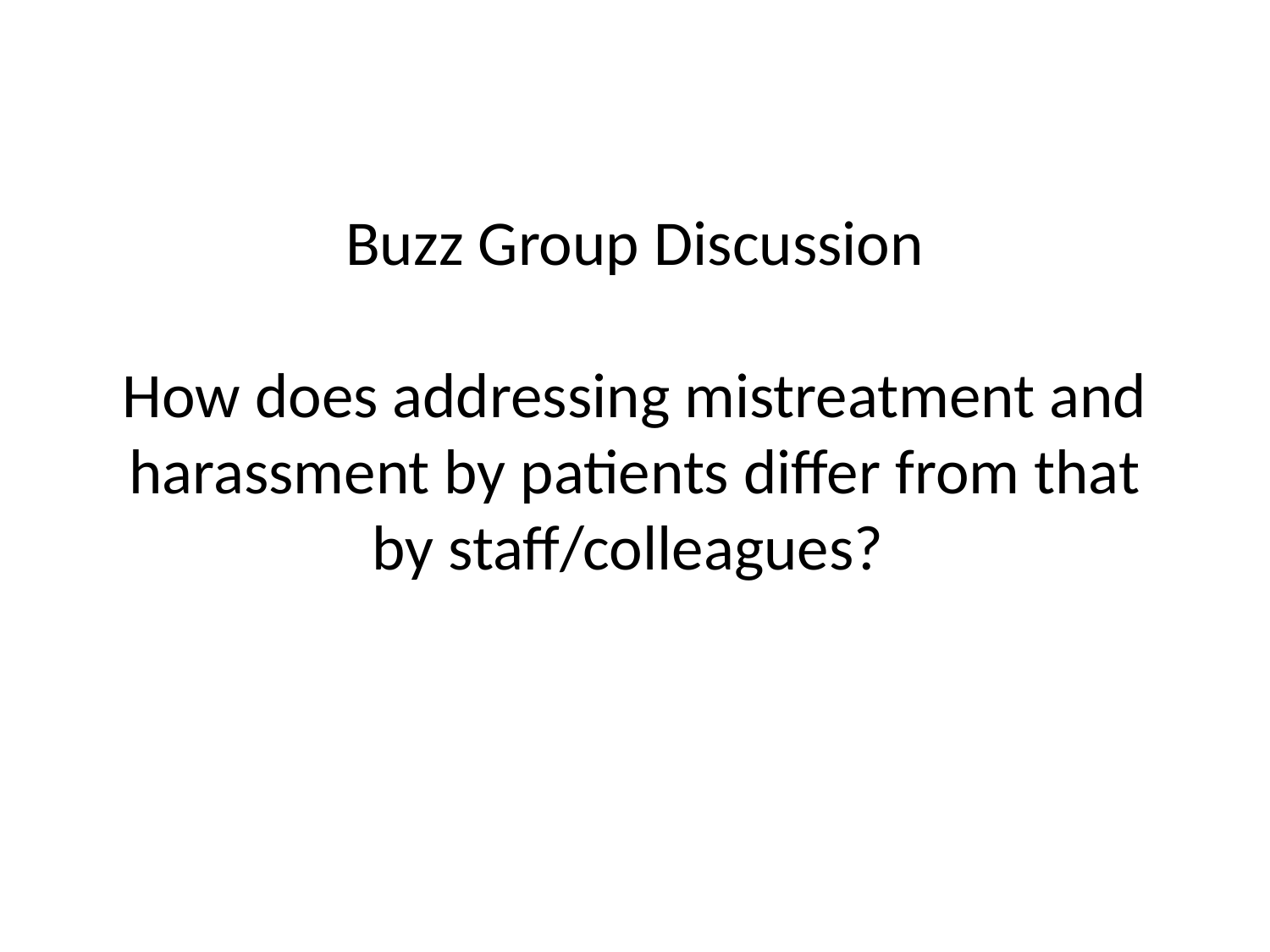

# Buzz Group Discussion How does addressing mistreatment and harassment by patients differ from that by staff/colleagues?

## Slide 15
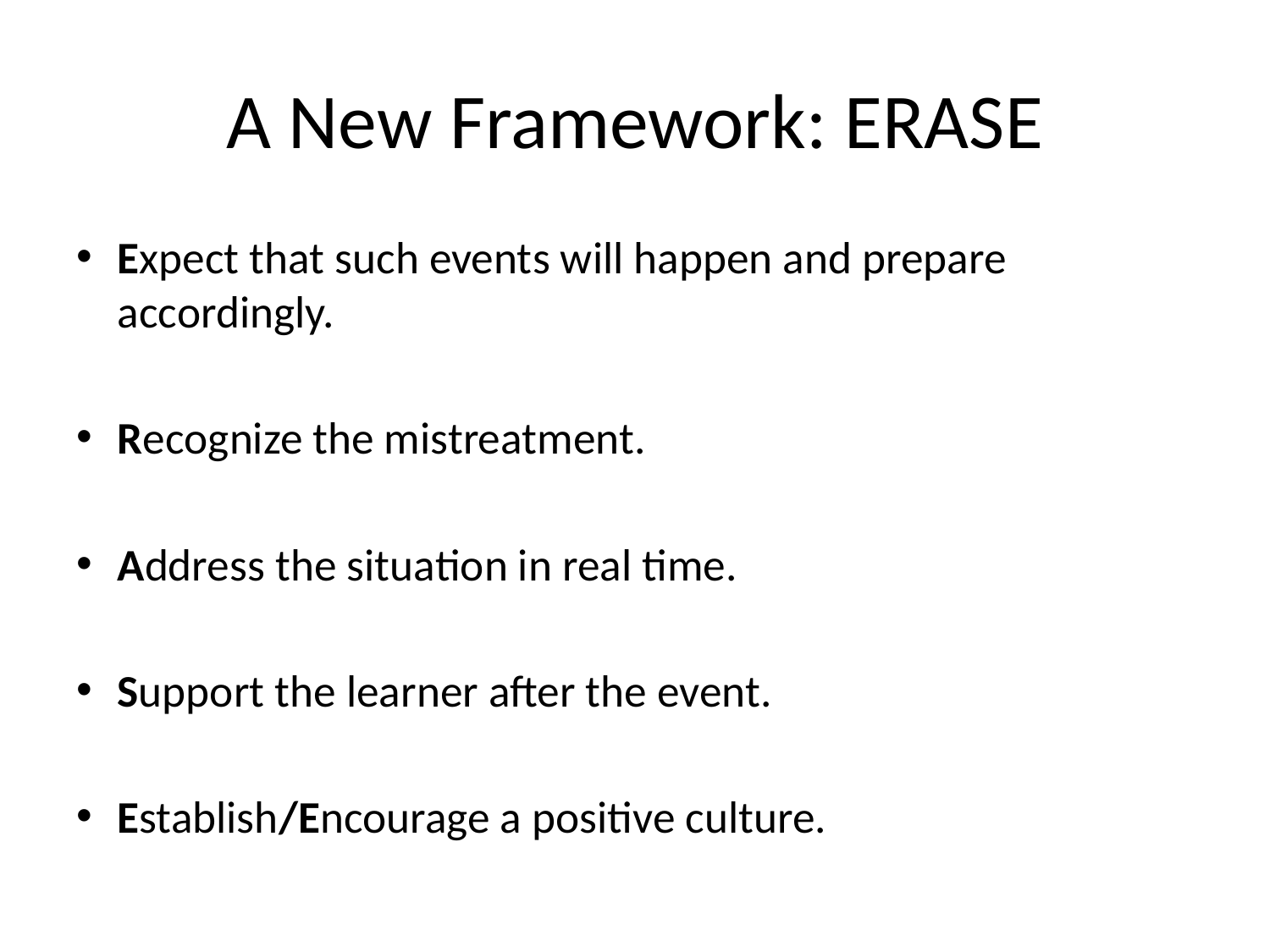

# A New Framework: ERASE
Expect that such events will happen and prepare accordingly.
Recognize the mistreatment.
Address the situation in real time.
Support the learner after the event.
Establish/Encourage a positive culture.

## Slide 16
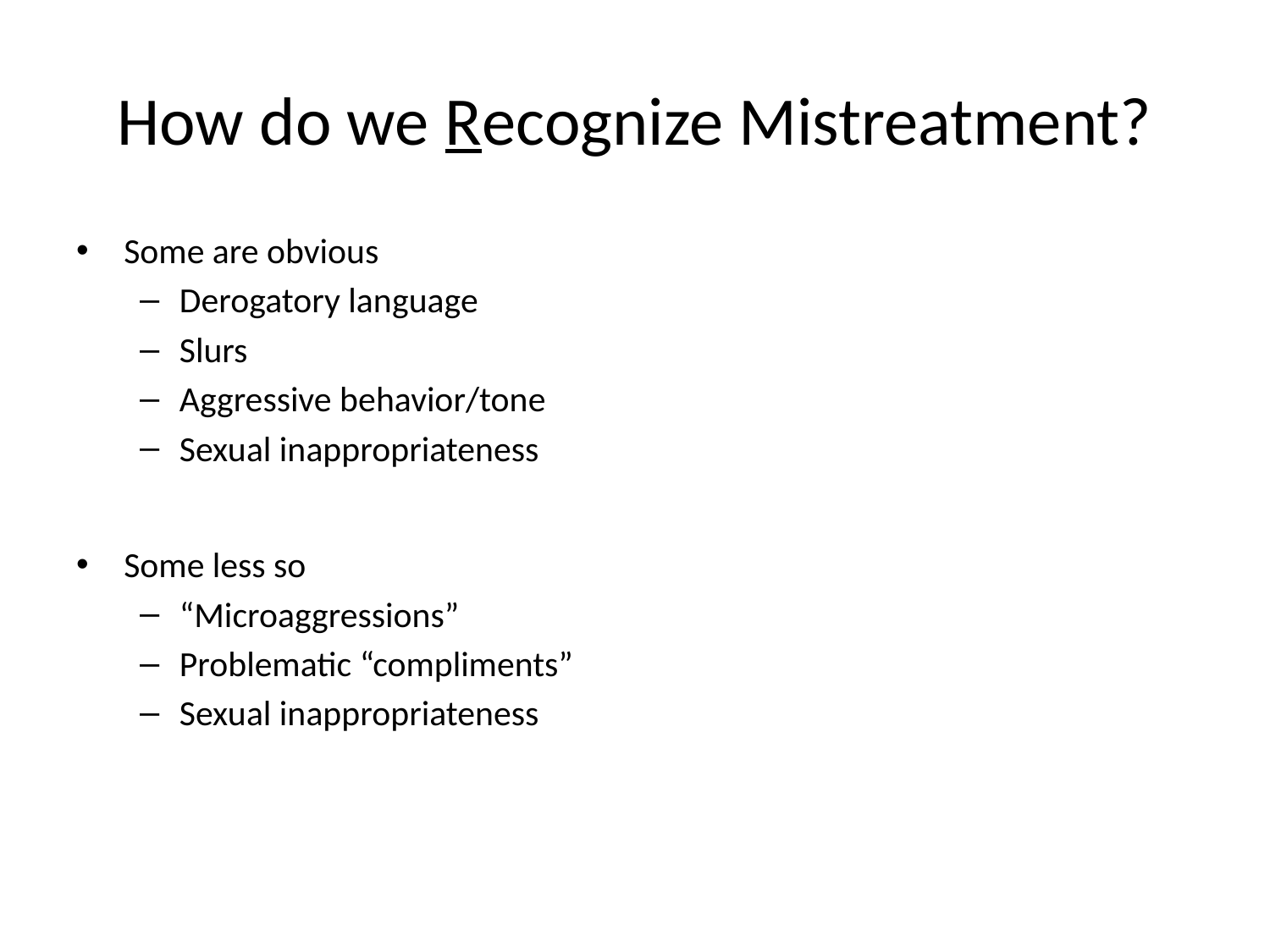

# How do we Recognize Mistreatment?
Some are obvious
Derogatory language
Slurs
Aggressive behavior/tone
Sexual inappropriateness
Some less so
“Microaggressions”
Problematic “compliments”
Sexual inappropriateness

## Slide 17
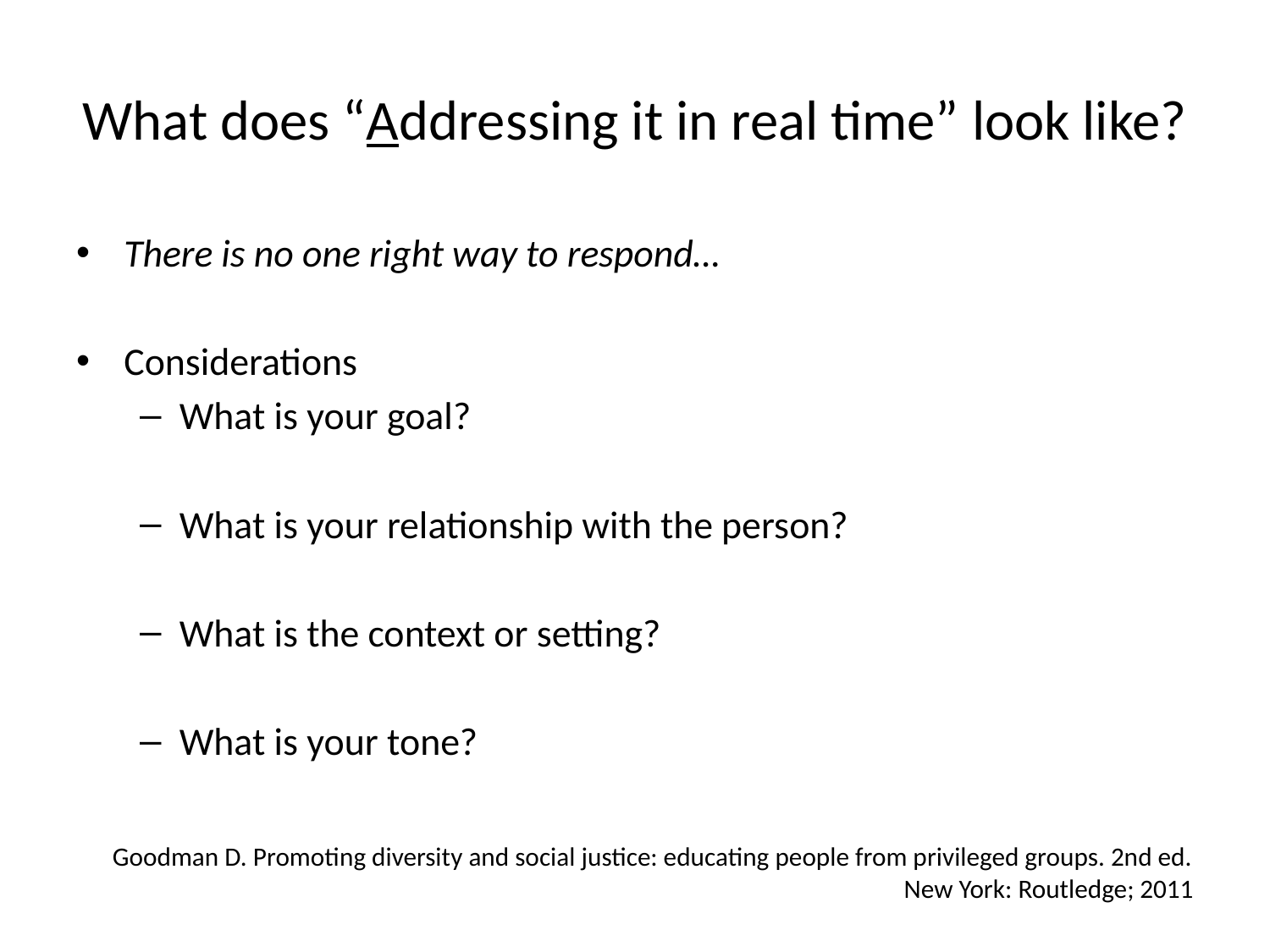

# What does “Addressing it in real time” look like?
There is no one right way to respond…
Considerations
What is your goal?
What is your relationship with the person?
What is the context or setting?
What is your tone?
Goodman D. Promoting diversity and social justice: educating people from privileged groups. 2nd ed. New York: Routledge; 2011

## Slide 18
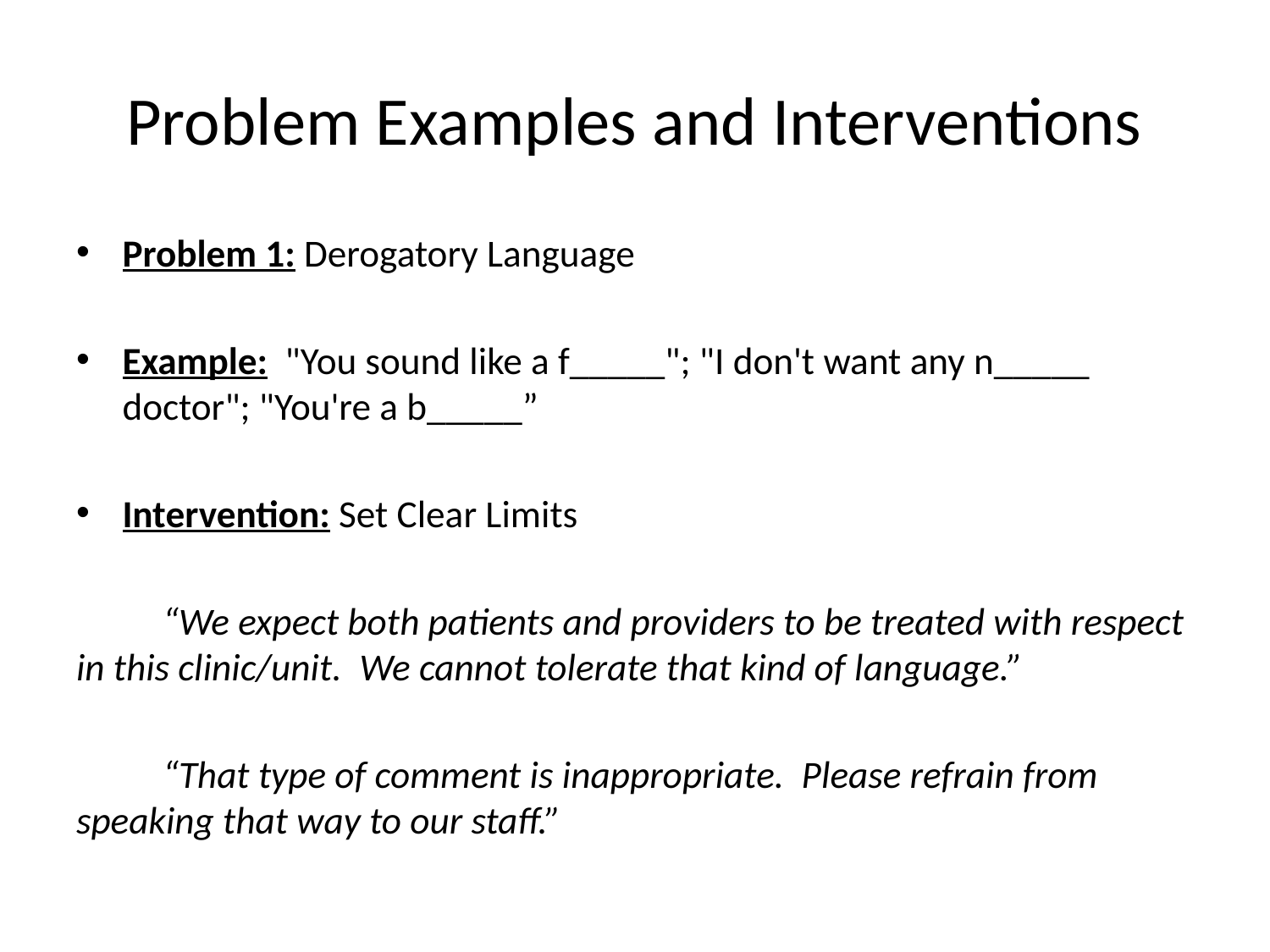

# Problem Examples and Interventions
Problem 1: Derogatory Language
Example:  "You sound like a f_____"; "I don't want any n_____ doctor"; "You're a b_____”
Intervention: Set Clear Limits
	“We expect both patients and providers to be treated with respect in this clinic/unit.  We cannot tolerate that kind of language.”
	“That type of comment is inappropriate. Please refrain from speaking that way to our staff.”

## Slide 19
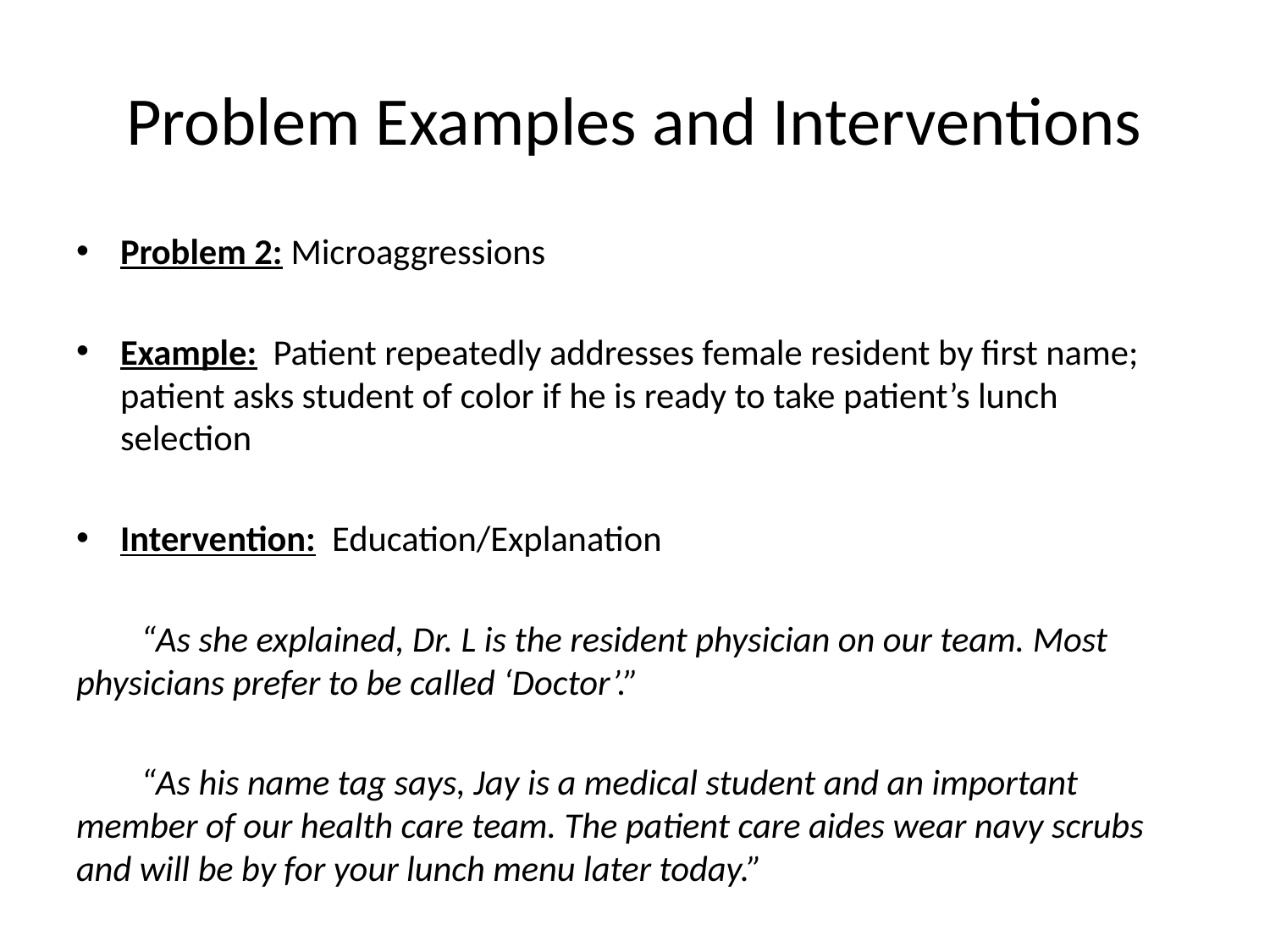

# Problem Examples and Interventions
Problem 2: Microaggressions
Example:  Patient repeatedly addresses female resident by first name; patient asks student of color if he is ready to take patient’s lunch selection
Intervention:  Education/Explanation
	“As she explained, Dr. L is the resident physician on our team. Most physicians prefer to be called ‘Doctor’.”
	“As his name tag says, Jay is a medical student and an important member of our health care team. The patient care aides wear navy scrubs and will be by for your lunch menu later today.”

## Slide 20
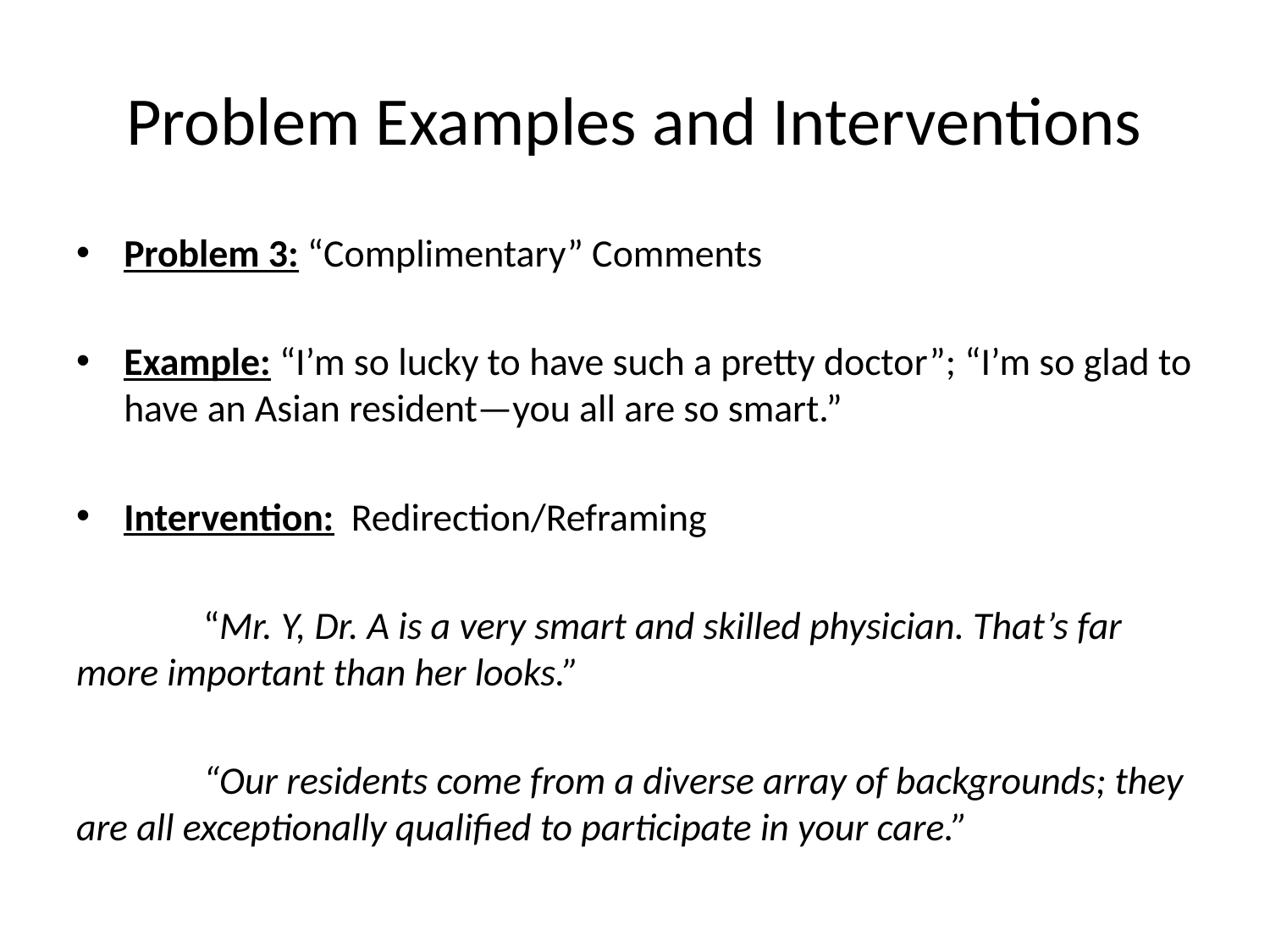

# Problem Examples and Interventions
Problem 3: “Complimentary” Comments
Example: “I’m so lucky to have such a pretty doctor”; “I’m so glad to have an Asian resident—you all are so smart.”
Intervention:  Redirection/Reframing
	“Mr. Y, Dr. A is a very smart and skilled physician. That’s far more important than her looks.”
	“Our residents come from a diverse array of backgrounds; they are all exceptionally qualified to participate in your care.”

## Slide 21
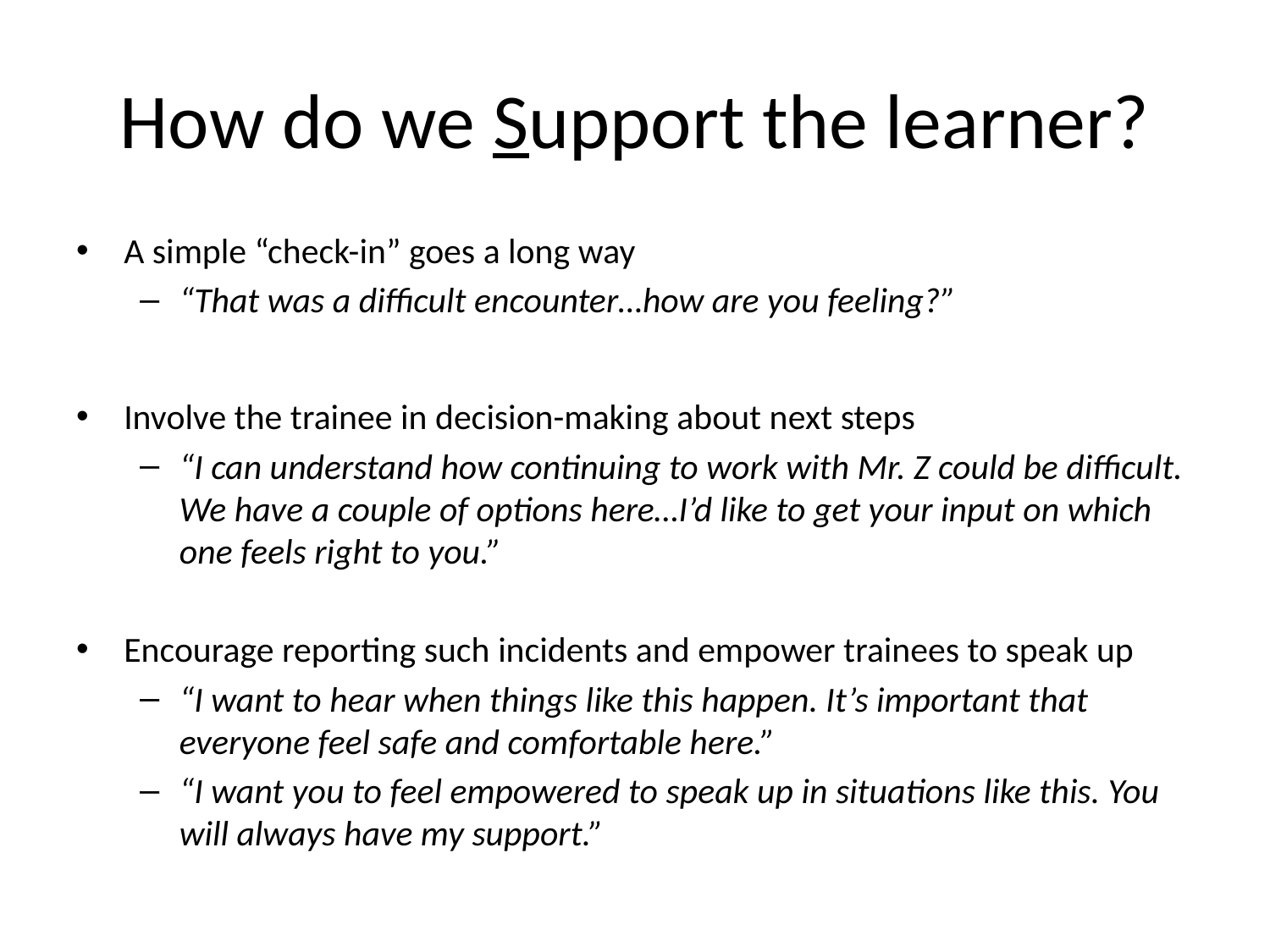

# How do we Support the learner?
A simple “check-in” goes a long way
“That was a difficult encounter…how are you feeling?”
Involve the trainee in decision-making about next steps
“I can understand how continuing to work with Mr. Z could be difficult. We have a couple of options here…I’d like to get your input on which one feels right to you.”
Encourage reporting such incidents and empower trainees to speak up
“I want to hear when things like this happen. It’s important that everyone feel safe and comfortable here.”
“I want you to feel empowered to speak up in situations like this. You will always have my support.”

## Slide 22
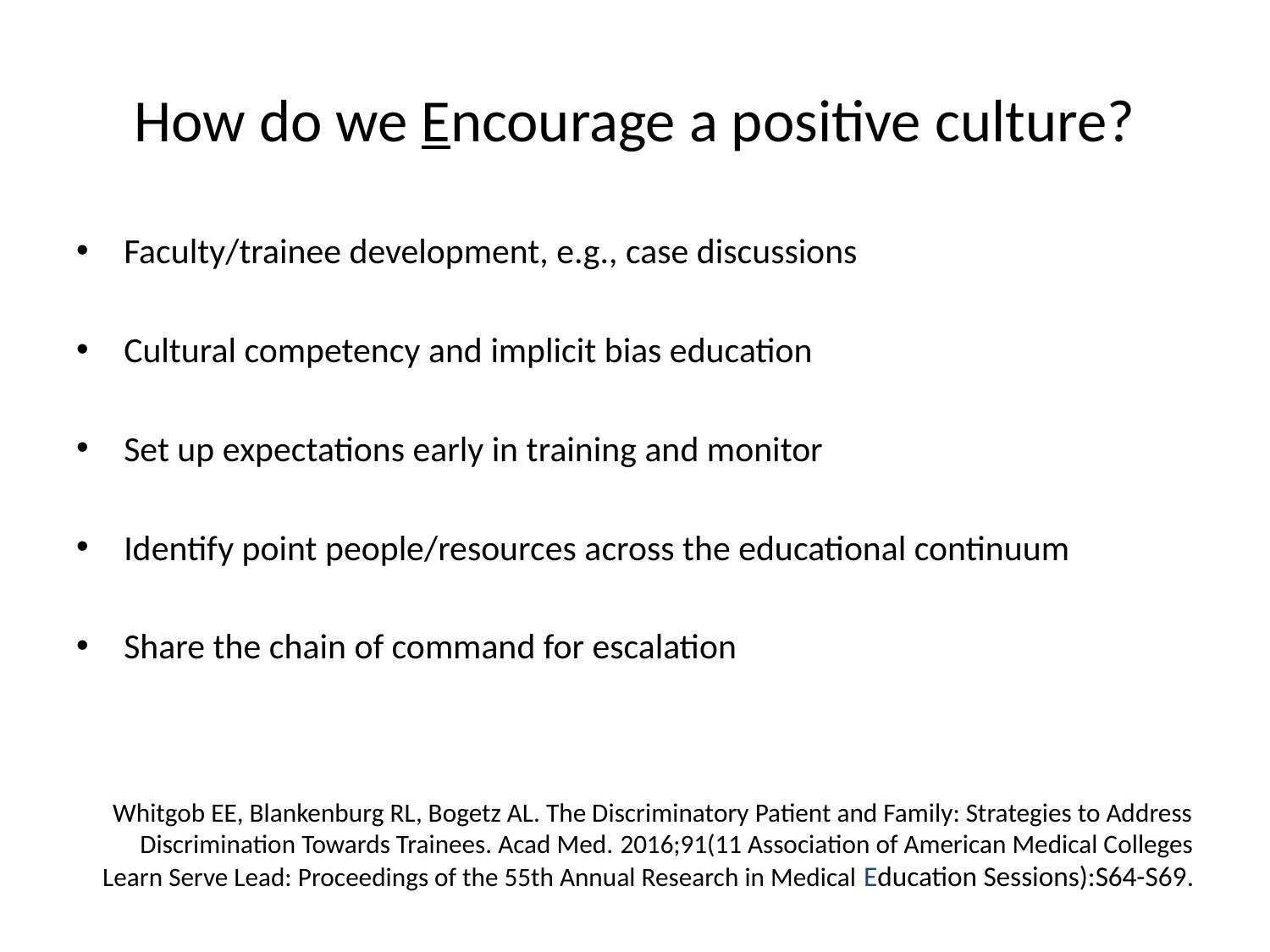

# How do we Encourage a positive culture?
Faculty/trainee development, e.g., case discussions
Cultural competency and implicit bias education
Set up expectations early in training and monitor
Identify point people/resources across the educational continuum
Share the chain of command for escalation
Whitgob EE, Blankenburg RL, Bogetz AL. The Discriminatory Patient and Family: Strategies to Address Discrimination Towards Trainees. Acad Med. 2016;91(11 Association of American Medical Colleges Learn Serve Lead: Proceedings of the 55th Annual Research in Medical Education Sessions):S64-S69.

## Slide 23
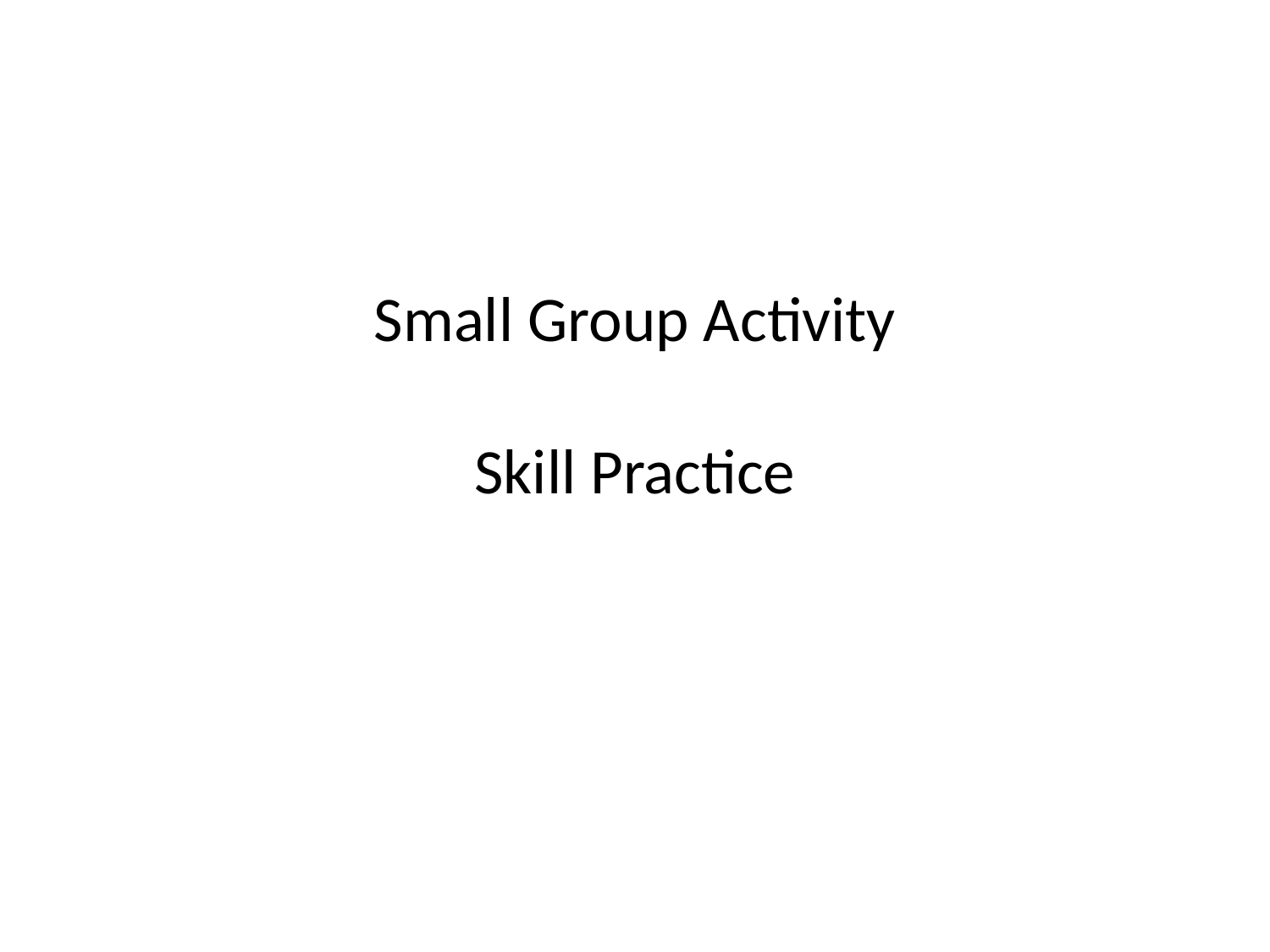

# Small Group Activity Skill Practice

## Slide 24
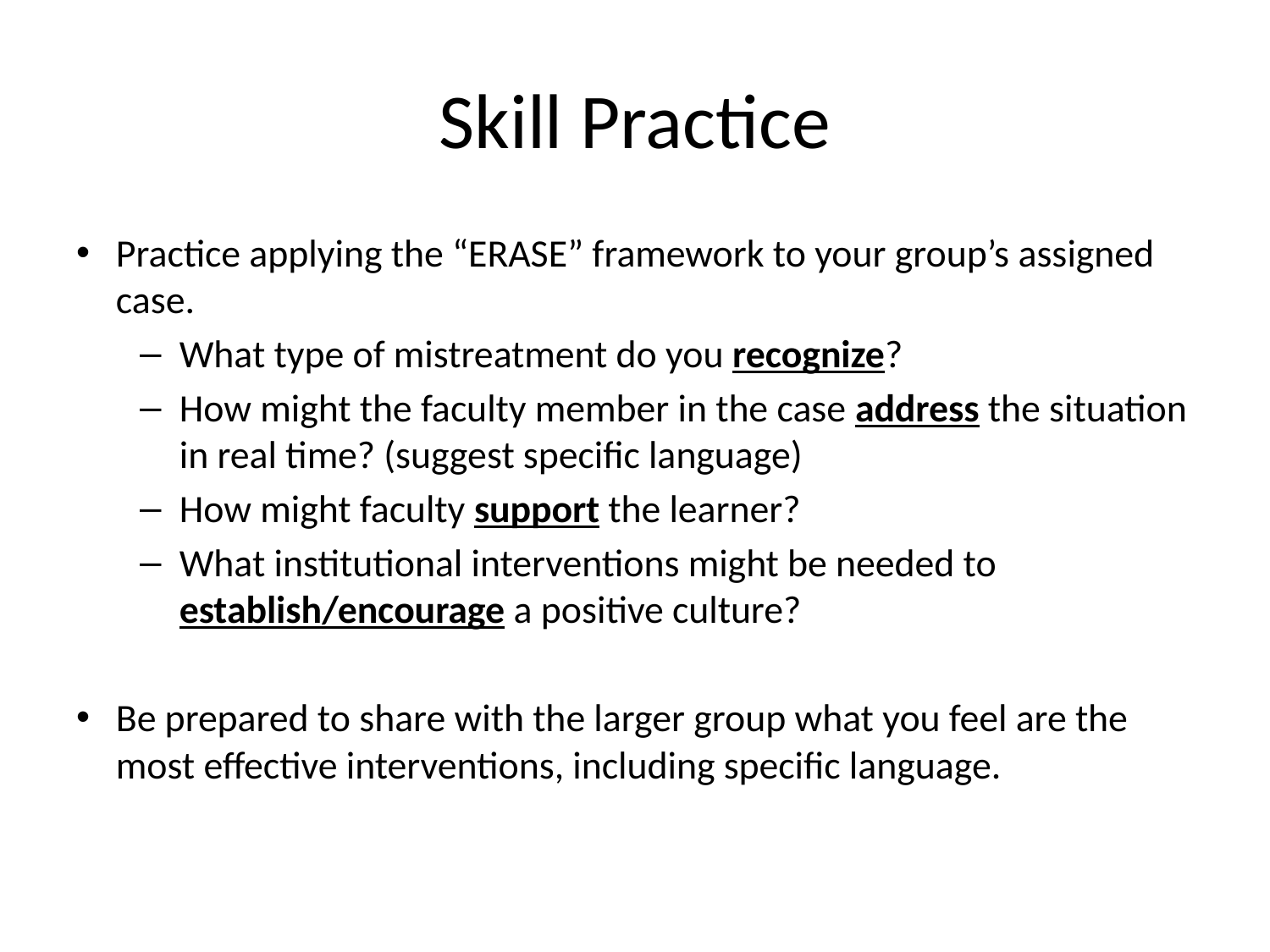

# Skill Practice
Practice applying the “ERASE” framework to your group’s assigned case.
What type of mistreatment do you recognize?
How might the faculty member in the case address the situation in real time? (suggest specific language)
How might faculty support the learner?
What institutional interventions might be needed to establish/encourage a positive culture?
Be prepared to share with the larger group what you feel are the most effective interventions, including specific language.

## Slide 25
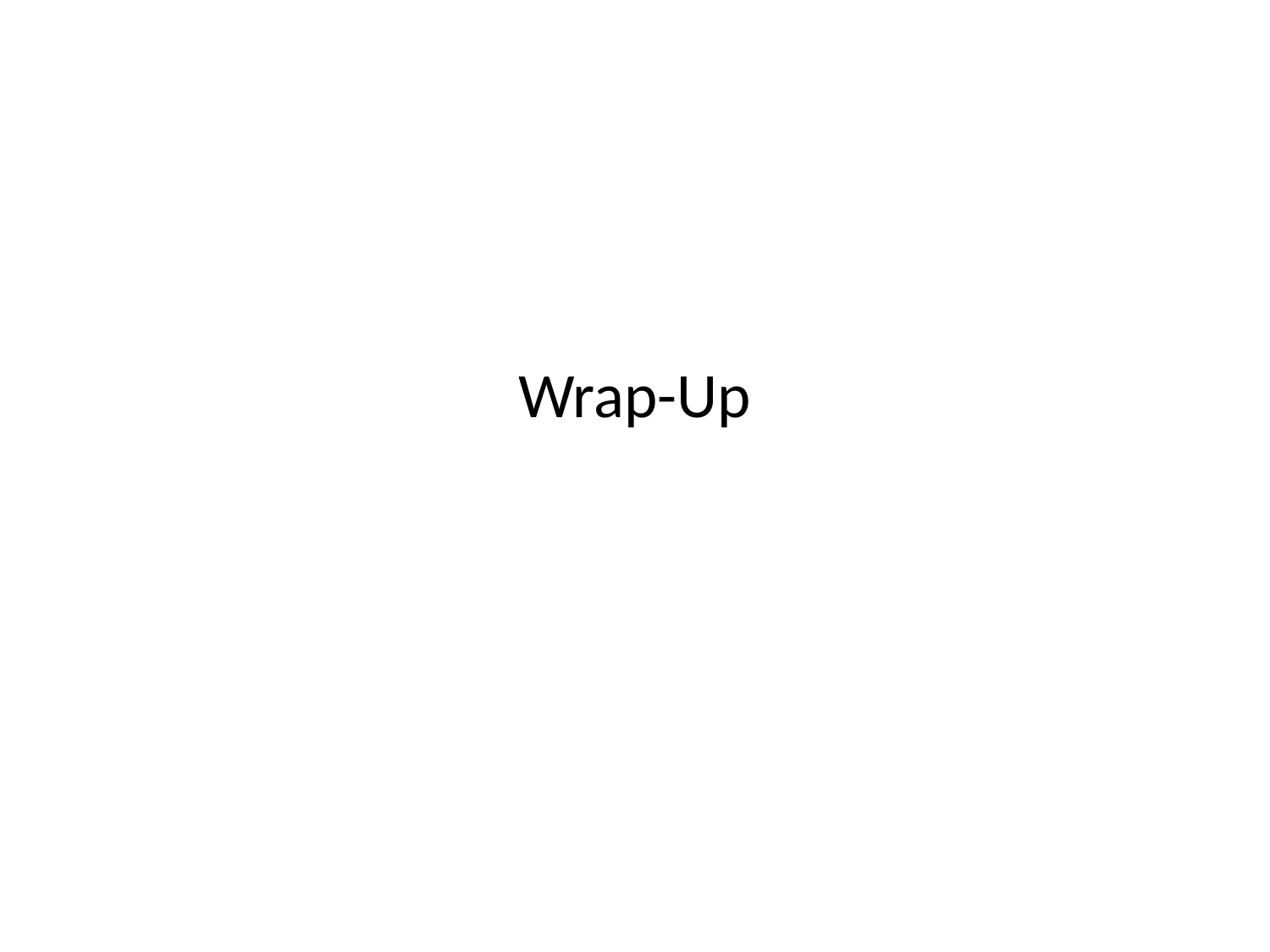

# Wrap-Up
