# Supplementary material for: ERASE-ing Patient Mistreatment of Trainees: Faculty Workshop
Source: MedEdPORTAL. 2019 Dec 27;15:10865. doi: 10.15766/mep_2374-8265.10865 (PMC7012314; doi:10.15766/mep_2374-8265.10865)
Supplement: Supplementary file 1 — A. Facilitator Guide.docx B. PowerPoint Presentation.pptx C. Case Examples.docx D. ERASE Model Handout.docx E. Available Resources and Reporting Mechanisms Handout.docx F. Pre- and Postsession Surveys.docx [file mep-15-10865-s001.zip › C. Case Examples.docx]

**ERASE-ing Mistreatment by Patients: Cases for Discussion**

***Instructions:*** *In each small group, assign roles and* ***discuss what should happen next****. Practice applying ERASE, with a focus on* ***recognizing, addressing, and supporting*** *the learners. Also consider whether any follow-up actions are needed to* ***establish/encourage a positive culture.*** *Groups will be asked to share with the larger group the interventions, including specific language, they believe to be the most effective.*

**Skill Application Case 1**

*Setting: inpatient service*

*A new team walks in to introduce themselves to the patient. The medical student is asked to take the lead.*

**Student:** “Hello, Mr. E. I’m Kris Whang, the medical student on your care team. This is Dr.…”

**Patient:** (looks at the student with disdain, then turns to the attending angrily) “Since when did you start hiring g**ks at this place? I don’t want any of them on my team!”

**Skill Application Case 2**

*Setting: outpatient clinic*

*Female resident has identified a unique finding on patient exam and has asked attending to come examine the patient.*

**Resident (addressing patient):** “Mr. S, I’d like to introduce you to Dr. Perez, the attending. I’ve given Dr. Perez all of your history and I’d like him to examine you as well.”

**Patient:** “Finally, a doctor…this nice nurse (gestures toward female resident) did such a thorough job, but I was wondering when I would finally get to see a doctor.”

**Skill Application Case 3**

*Setting: Outpatient clinic, follow up visit for AZ, a longtime patient of the attending*

*Student Taylor has evaluated AZ and is returning to see the patient with the attending.*

**Attending:** “AZ, it’s good to see you again. Taylor has been telling me about your symptoms. How are you feeling?”

**AZ:** “Not so good, Doc. But I have to say, talking to Taylor sure made me feel better. How did you get so lucky to have such a good-looking med student? How do you get any work done around here?”

**Taylor:** (looks embarrassed, uncomfortable)

**Additional Skill Application Case**

*Setting: Waiting area of outpatient clinic*

*A patient is running late for their appointment and has been waiting in a long line to check in. The office is short staffed and the patient is increasingly irritable. The attending and student come to the waiting room to escort the patient to the attending’s office.*

**Attending:** “Hello, Ms. B. Since we’re already running behind, let’s head over to my office.”

**Patient (raised voice):** “Well, I would have been on time if these f&#&ing idiots at the front desk knew what they were doing! If it were up to me, they’d just go back to their own country!”

**Additional Skill Application Case**

*Setting: outpatient clinic*

*Student is interviewing a patient in front of attending.*

**Student:** “Ms. W, now I’d like to ask you some questions about your background and your family history…”

**Patient:** “Okay, but first let me ask you a question about your background. Where are you from?”

**Student:** “Um…I’m from New Jersey…now let’s get back to your history…”

**Patient (laughs):** “No, c’mon kid, where are you **really** from?”

**Additional Skill Application Case**

*Setting: inpatient service*

*Attending and resident walk in to introduce themselves to a patient.*

**Resident:** “Hi, Mr. Q, I’m Dr. B. It’s nice to meet you.”

**Veteran:** “Oh thank God, you don’t have an accent. When I heard your name from the nurse, I wasn’t sure. I’m glad I got a doctor who speaks good English!”

**Additional Skill Application Case**

*Setting: outpatient resident clinic*

*Resident brings an elderly patient with their adult son back to the office. Attending is present to observe and provide feedback on the resident interview and exam.*

**Patient to Resident: “**Doc, it’s always so good to see you. I feel better the minute I see you. See, son, what did I tell you? She’s gorgeous!”

**Patient’s Son:** “Aww, she’s blushing.”

**Resident** (looks embarrassed, uncomfortable)
