# Supplementary material for: ERASE-ing Patient Mistreatment of Trainees: Faculty Workshop
Source: MedEdPORTAL. 2019 Dec 27;15:10865. doi: 10.15766/mep_2374-8265.10865 (PMC7012314; doi:10.15766/mep_2374-8265.10865)
Supplement: Supplementary file 1 — A. Facilitator Guide.docx B. PowerPoint Presentation.pptx C. Case Examples.docx D. ERASE Model Handout.docx E. Available Resources and Reporting Mechanisms Handout.docx F. Pre- and Postsession Surveys.docx [file mep-15-10865-s001.zip › D. ERASE Model Handout.docx]

**“ERASE” Framework for Managing Trainee Mistreatment by Patients**

**E**xpect that such events will happen and prepare accordingly.

-Attend workshop

-Talk/Read/Rehearse specific language

-Provide anticipatory guidance to trainees

**R**ecognize the mistreatment.

-Consider perspective of trainee

-Pay particular attention to potential microaggressions, “compliments”

**A**ddress the situation in real time. *

-Distinguish between types of mistreatment

-Use specific language/technique in different situations

-Practice speaking up

***There is no one right way to respond!**

**Consider…**

-What is your goal?

-What is your relationship with the person?

-What is the context or setting?

-What is your tone?

Goodman D. Promoting diversity and social justice: educating people from privileged groups. 2nd ed. New York: Routledge; 2011**.**

**S**upport the learner after the event.

-Ask trainee how they experienced event

-Listen and respond to concerns

-Engage in decision-making about next steps

**E**stablish/encourage a positive culture.

-Express openness to hearing concerns

-Develop and disseminate reporting mechanism

-Consider policies, signage regarding non-tolerance for mistreatment

**Problem Examples and Interventions**

**Problem 1:** **Macroaggressions/Derogatory Statements**

**Example:**  "You sound like a f_____"; "I don't want any n_____ doctor"; "You're a b_____"

**Intervention:** Set Clear Limits

*“We expect both patients and providers to be treated with respect in this clinic/unit.  We cannot tolerate that kind of language.”*

*“That type of comment is inappropriate. Please refrain from speaking that way to our staff.”*

**Problem 2:** **Microaggressions**

**Example:**  Patient repeated addresses female resident by first name; patient asks student of color if he is ready to take patient’s lunch selection

**Intervention:**  Education/Explanation

*"As she explained, Dr. L is the resident physician on our team. Most physicians prefer to be called ‘Doctor’.”*

*“As his name tag says, Jay is a medical student and an important member of our health care team. The patient care aides wear navy scrubs and will be by for your lunch menu later today.”*

**Problem 3:** **“Complimentary” Comments**

**Example:** “I’m so lucky to have such a pretty doctor”; “I’m so glad to have an Asian resident—they are always so smart.”

**Intervention:**  Redirection/Reframing

“*Mr. Y, Dr. A is a very smart and skilled physician. That’s far more important than her looks.”*

*“Our residents come from a diverse array of backgrounds; they are all exceptionally qualified to participate in your care.”*
