# Supplementary material for: ERASE-ing Patient Mistreatment of Trainees: Faculty Workshop
Source: MedEdPORTAL. 2019 Dec 27;15:10865. doi: 10.15766/mep_2374-8265.10865 (PMC7012314; doi:10.15766/mep_2374-8265.10865)
Supplement: Supplementary file 1 — A. Facilitator Guide.docx B. PowerPoint Presentation.pptx C. Case Examples.docx D. ERASE Model Handout.docx E. Available Resources and Reporting Mechanisms Handout.docx F. Pre- and Postsession Surveys.docx [file mep-15-10865-s001.zip › E. Available Resources and Reporting Mechanisms Handout.docx]

**Available Resources and Reporting Mechanisms for Mistreatment by Patients**

- Clinical faculty and site training directors
- Chief Residents: Program-Wide and Chief Residents for Diversity
- Clerkship Director (for student-related concerns)
- Program Director and Associate Program Directors (for resident-related concerns)
- Assistant Chair for Diversity and Departmental Diversity Task Force
- Resident and student tutors
- Ombudsperson
- Title IX Coordinators
- LGBTQ Office
- Anonymous end of rotation evaluation forms
- Computerized adverse event reporting system
