# Supplementary material for: ERASE-ing Patient Mistreatment of Trainees: Faculty Workshop
Source: MedEdPORTAL. 2019 Dec 27;15:10865. doi: 10.15766/mep_2374-8265.10865 (PMC7012314; doi:10.15766/mep_2374-8265.10865)
Supplement: Supplementary file 1 — A. Facilitator Guide.docx B. PowerPoint Presentation.pptx C. Case Examples.docx D. ERASE Model Handout.docx E. Available Resources and Reporting Mechanisms Handout.docx F. Pre- and Postsession Surveys.docx [file mep-15-10865-s001.zip › F. Pre- and Postsession Surveys.docx]

**Pre-Session Survey for Trainee Mistreatment Workshop**

|  |  | **Yes** | | **No** | | | **Unsure** | |
| --- | --- | --- | --- | --- | --- | --- | --- | --- |
| 1 | Prior to today’s session, have you received specific training in how to recognize and address episodes of trainee mistreatment? |  | |  | | |  | |
|  |  | **Strongly Disagree** | **Disagree** | | **Neutral** | **Agree** | | **Strongly Agree** |
| 2 | Mistreatment of health care providers, including trainees, by patients is a common experience which can have a significant psychological impact. |  |  | |  |  | |  |
| 3 | I intervene effectively when I learn of an episode of mistreatment. |  |  | |  |  | |  |
| 4 | I foster a psychologically safe learning environment for trainees. |  |  | |  |  | |  |
| 5 | It is my role to intervene when I learn that a trainee under my supervision has been mistreated by a patient. |  |  | |  |  | |  |
| 6 | It is my role to intervene when I learn that a trainee under my supervision has been mistreated by another member of the healthcare team. |  |  | |  |  | |  |
| 7 | I can recognize multiple forms of trainee mistreatment, including abuse, harassment and microaggressions. |  |  | |  |  | |  |
| 8 | I know how to intervene when I learn of an episode of trainee mistreatment by patients. |  |  | |  |  | |  |
| 9 | I know how to intervene when I learn of an episode of trainee mistreatment by other members of the healthcare team. |  |  | |  |  | |  |
| 10 | I have a standardized approach I can utilize when addressing an episode of trainee mistreatment. |  |  | |  |  | |  |

**Post-Session Survey for Trainee Mistreatment Workshop**

|  |  | **Yes** | | **No** | | | **Unsure** | |
| --- | --- | --- | --- | --- | --- | --- | --- | --- |
| 1 | Prior to today’s session, have you received specific training in how to recognize and address episodes of trainee mistreatment? |  | |  | | |  | |
|  |  | **Strongly Disagree** | **Disagree** | | **Neutral** | **Agree** | | **Strongly Agree** |
| 2 | Mistreatment of health care providers, including trainees, by patients is a common experience which can have a significant psychological impact. |  |  | |  |  | |  |
| 3 | I intervene effectively when I learn of an episode of mistreatment. |  |  | |  |  | |  |
| 4 | I foster a psychologically safe learning environment for trainees. |  |  | |  |  | |  |
| 5 | It is my role to intervene when I learn that a trainee under my supervision has been mistreated by a patient. |  |  | |  |  | |  |
| 6 | It is my role to intervene when I learn that a trainee under my supervision has been mistreated by another member of the healthcare team. |  |  | |  |  | |  |
| 7 | I can recognize multiple forms of trainee mistreatment, including abuse, harassment and microaggressions. |  |  | |  |  | |  |
| 8 | I know how to intervene when I learn of an episode of trainee mistreatment by patients. |  |  | |  |  | |  |
| 9 | I know how to intervene when I learn of an episode of trainee mistreatment by other members of the healthcare team. |  |  | |  |  | |  |
| 10 | I have a standardized approach I can utilize when addressing an episode of trainee mistreatment. |  |  | |  |  | |  |
